# Developing an erythrocyte‒MHC-I conjugate for cancer treatment

**DOI:** 10.1038/s41421-024-00713-9

**Published:** 2024-10-01

**Authors:** Yuehua Liu, Xiaoqian Nie, Xingyun Yao, Huafeng Shou, Yang Yuan, Yun Ge, Xiangmin Tong, Hsiang-Ying Lee, Xiaofei Gao

**Affiliations:** 1 Zhejiang University, School of Basic Medical Science, Hangzhou, Zhejiang China; 2 Key Laboratory of Growth Regulation and Translational Research of Zhejiang Province, School of Life Sciences, Westlake University, Hangzhou, Zhejiang China; 3 Westlake Laboratory of Life Sciences and Biomedicine, Hangzhou, Zhejiang China; 4 Institute of Basic Medical Sciences, Westlake Institute for Advanced Study, Hangzhou, Zhejiang China; 5 Department of Gynecology, Zhejiang Provincial People’s Hospital, Hangzhou, Zhejiang China; 6 Westlake Therapeutics Co., Ltd., Hangzhou, Zhejiang China; 7 Department of Hematology, Zhejiang Provincial People’s hospital, Hangzhou, Zhejiang China; 8 Ministry of Education Key Laboratory of Cell Proliferation and Differentiation, School of Life Sciences, Peking University, Beijing, China

**Keywords:** Cancer immunotherapy, Immunology

## Abstract

Mature erythrocytes are known to lack major histocompatibility complex (MHC) proteins. However, the presence of MHC molecules on erythrocytes has been occasionally reported, though without a defined function. In this study, we designed erythrocyte conjugated solely with a fusion protein consisting of an antigenic peptide linked to MHC class I (MHC-I) protein, termed MHC-I‒Ery. The modified erythrocyte, decorated with the peptide derived from human papillomavirus (HPV) 16 oncoprotein E6/E7, effectively activated antigen-specific CD8^+^ T cells in peripheral blood mononuclear cells (PBMCs) from HPV16^+^ cervical cancer patients. Additionally, MHC-I‒Ery monotherapy was shown to inhibit antigen-positive tumor growth in mice. This treatment immediately activated CD8^+^ T cells and reduced suppressive myeloid cells in the spleen, leading to systemic anti-tumor activity. Safety and tolerability evaluations of MHC-I‒Ery in non-human primates further supported its clinical potential. Our results first demonstrated that erythrocytes equipped solely with antigen peptide‒MHC-I complexes can robustly stimulate the immune system, suggesting a novel and promising approach for advancing cancer immunotherapy.

## Introduction

Erythrocytes are the most abundant cells in the human body, making up about 70% of an adult’s total cell count. Traditionally recognized for their role in oxygen transport, erythrocytes are uniquely suited to serve as drug delivery vehicles due to their lack of nuclei, biocompatibility, high surface area-to-volume ratio, and membrane flexibility^1,2^. These properties not only ensure their safety over long durations but also make them ideal for ferrying various therapeutics^3,4^. In addition to their well-known functions, recent findings have highlighted a significant role for erythrocytes in modulating the immune system^5^. Research has consistently shown that erythrocytes possess the capacity to bind and interact with various inflammatory molecules, such as chemokines, nucleic acids, and pathogens. For example, the Duffy antigen receptor for chemokines (DARC) on erythrocytes can act as a sink for CXCL8, thereby inactivating the CXCL8-dependent chemokine gradient and preventing neutrophil recruitment^6^. In addition to CXCL8, Duffy also exhibits high affinity for other immune-regulating proteins, including additional chemokines^7,8^. This ability allows erythrocytes to effectively regulate and modulate immune responses. Through the direct cell‒cell interaction, erythrocytes can inhibit the formation of protein-bound acrolein, a marker of oxidative stress, generated during in vitro T cell activation^9^.

Within mammals, the spleen, the largest secondary lymphoid organ, plays a crucial role in coordinating immune responses both in health and disease conditions^10^. It contains approximately one-third of all immune cells and presents the highest concentration of antigen-presenting cells (APCs) for priming T cells^11–13^. However, during tumor progression, the spleen can harbor suppressive myeloid cells that dampen T cell activation and contribute to tumorigenesis^14,15^. The mononuclear phagocyte system (MPS) within the spleen also poses a challenge to drug delivery, adeptly clearing biological drugs through phagocytosis, limiting medication efficacy^16^. Interestingly, erythrocytes cross the spleen every 20 min, providing frequent opportunities for interaction with immune cells^17^.

It is well-known that mature mammalian erythrocytes commonly lack major histocompatibility complex (MHC) proteins and human leukocyte antigen (HLA) matching is typically not a standard consideration for blood transfusion^18,19^. However, erythrocytes are capable of expressing antigen-presenting molecules under certain pathological conditions^20^. This phenomenon is observed in erythrocytes infected by the malaria parasite, where MHC molecules can trigger CD8^+^ T cell recognition, leading to phosphatidylserine (PS) externalization and phagocytosis of the affected erythrocytes^21^. Moreover, an increase in MHC-I expression on erythrocytes has been documented in patients with systemic lupus erythematosus, correlating with disease severity^22^. These observations suggest a contributory role for erythrocytes in immune regulation, although their exact impact on immune cell activity remains to be fully elucidated. Previous studies have explored the role of peptide‒MHC fusion proteins together with other molecules on erythrocytes in the modulation of immune response for treating cancer, while the efficacy with only MHC-I molecules remains unknown^23–25^. For instance, Zhang et al. have genetically engineered erythrocytes differentiated from hematopoietic stem cells (HSCs) expressing 4-1BBL (4-1BB ligand) and Interleukin 12 (IL-12) along with MHC-I molecules^24^. However, anti-tumor activities of these engineered erythrocytes are dependent upon the function of 4-1BBL and IL-12, with the contribution of conjugated MHC-I molecules being very limited. Additionally, erythrocytes have been modified to present peptide‒MHC-I complexes and anti-CD28 antibodies with DNA linkers^25^. However, the efficacy of these modified erythrocytes in activating endogenous antigen-specific T cells in tumor-bearing hosts is limited without co-administration of exogeneous antigen-specific T cells^25^. This could be due to low drug payloads and potential erythrocyte damage during modification, leading to reduced lifespan and limited therapeutic effectiveness, with only 10% of engineered erythrocytes surviving two days after infusion^23^. Moreover, the utilization of a DNA-directed assembly strategy has been previously found to result in cell membrane disturbance, damage, and cell death^26,27^. Thus, there is still a question regarding the efficacy of erythrocytes bearing solely MHC-I molecules in immune activation.

We have previously developed an innovative strategy by conjugating molecules on erythrocyte membranes^28^. This conjugation, achieved through a sortase A variant (mgSrtA) enzyme-mediated reaction, preserves the natural characteristics of erythrocytes, as evidenced by the similar biological characteristics and comparable survival of engineered erythrocytes to natural ones. Meanwhile, these engineered erythrocytes showed therapeutic efficacy in disease models. In this study, by using this conjugation technology, we designed a novel type of therapeutic erythrocytes by conjugating human papillomavirus (HPV) 16 oncoprotein E6 or E7 (E6/E7) peptide-loaded HLA-A*02:01 fused with the fragment crystallizable (Fc) region of the human immunoglobulin class G1 (IgG1 Fc) onto erythrocyte membranes, which were termed MHC-I‒E6/E7‒Ery. We demonstrated that these MHC-I‒E6/E7‒Ery successfully activated T cells and enhanced cytotoxic responses against tumor cells in vitro, using peripheral blood mononuclear cells (PBMCs) from HPV16^+^ cervical cancer patients. Moreover, MHC-I‒E6/E7‒Ery substantially increased the expansion of antigen-specific CD8^+^ T cells and reduced the reservoir of suppressive myeloid cells in the spleen. These changes further reshaped the tumor microenvironment (TME) by decreasing suppressive myeloid cells and enhancing tumor-specific T cell infiltration, resulting in significant inhibition of tumor growth in animal models. When combined with anti-PD-1 antibody treatment, MHC-I‒E6/E7‒Ery exhibited synergistic therapeutic effects. Safety and pharmacokinetics assessments in cynomolgus macaques support further development of this innovative therapy. Collectively, our results for the first time demonstrated that erythrocytes equipped with antigen peptide‒MHC-I complexes alone can efficiently stimulate the immune system, offering a promising novel clinical strategy for cancer treatment.

## Results

### Erythrocytes conjugated with mouse MHC-I molecules efficiently activate T cells

**Fig. 1:**
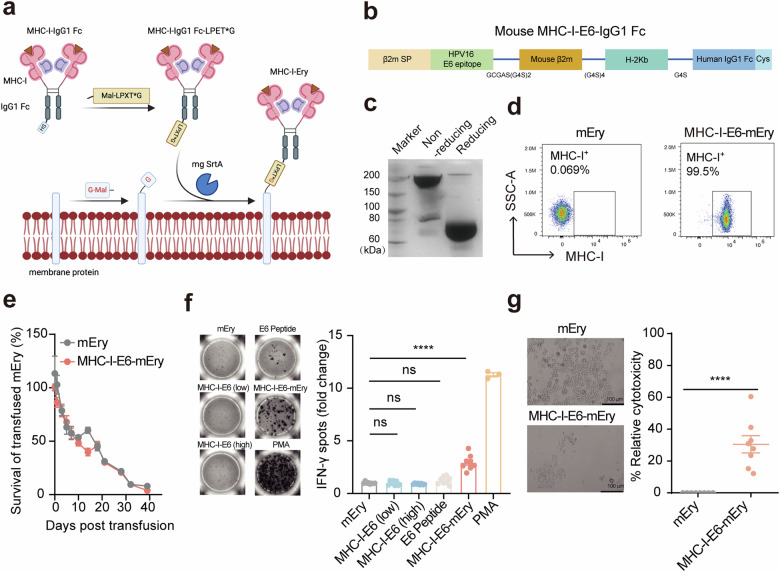
Erythrocytes conjugated with MHC-I molecules efficiently activate T cells in vitro*.* **a** Schematic representation of the MHC-I‒Ery preparation procedure. G-Mal: GAASK-6-maleimide. Mal-LPET*G: the asterisk denotes the 2-hydroxyacetic acid between the threonine and glycine residues. **b** Diagram illustrating the mouse MHC-I‒E6‒IgG1 Fc construct. **c** SDS-PAGE analysis of the purified mouse MHC-I‒E6‒IgG1 Fc fusion proteins. **d** Flow cytometry plots of MHC-I‒E6‒mEry stained for human IgG. **e** Pharmacokinetic study of MHC-I‒E6‒mEry and mEry in C57BL/6 mice. Far-Red-labeled MHC-I‒E6‒mEry or mEry were injected into C57BL/6 mice intraveneously (i.v.) Blood samples were collected at the specified times, and the percentage of Far-Red-labeled erythrocytes in circulation were determined by flow cytometry (*n* = 5 mice per group). **f** IFN-γ secreting T cells were assessed using an ELISpot assay. Splenocytes harvested from MC38-HPV16 tumor-bearing mice were co-incubated with mEry, MHC-I‒E6‒mEry, HPV16 E6 peptide (KCLKFYSKI) or recombinant protein of MHC-I‒E6 for 48 h. Low (0.5 μg/mL) or high (25 μg/mL) represent recombinant MHC-I‒E6 protein dosages used in this experiment. Left: representative images of ELISpot wells. Right: the quantification of IFN-γ spots (*n* = 8 per group, except for *n* = 3 for PMA group). **g** In vitro killing assay. Splenocytes from MC38-HPV16 tumor-bearing mice were cultured with mEry or MHC-I‒E6‒mEry for 48 h. Then, CD8^+^ T cells were isolated and further co-cultured with Calcein AM-labeled MC38-HPV16 tumor cells for additional 72 h. The survival of the MC38-HPV16 tumor cells were subsequently calculated by Calcein AM fluorescence. Left: representative images of tumor cells after the killing assay. Right: the relative cytotoxicity of CD8^+^ T cells against tumor cells upon different treatments (*n* = 8 per group). Data are presented as means ± SEM. Significance was determined by one-way analysis of variance (ANOVA) with Dunnett’s multiple-comparison test (**f**) or unpaired *t*-test (**g**). The significance levels are indicated as follows: ns, not significant; ****P < 0.0001.

We initially explored the feasibility of generating erythrocyte‒MHC-I conjugates. HPV-associated malignancies account for approximately 4.5% of all cancers, affecting over 600,000 individuals annually^29^. These cancers span anal, cervical, oropharyngeal, penile, vaginal, and vulvar cancer, with recurrent HPV^+^ cancers exhibiting poor survival rates^30^. Focusing on the immunologically targetable E6 and E7 oncoproteins of HPV, prevalent in many cervical cancer patients, we concentrated on epitopes known to induce CD8^+^ T cell responses^31^. The HPV type 16-E7 epitope (YMLDLQPET), recognized by HLA-A*02:01, is frequently identified in cervical biopsies from cancer patients, while the HPV type 16-E6 epitope (KCLKFYSKI) is recognized by H-2Kb, known for eliciting strong T cell reactions in mice^32,33^. We recently reported a novel enzyme-mediated conjugation method on natural erythrocytes^28^. Specifically, we employed a sortase variant known as mg SrtA, which selectively cleaves the threonine-glycine within a protein containing the LPXTG motif (protein A). This cleavage generates an acyl intermediate between the mg SrtA’s active site cysteine residue (HS) and the threonine residue in protein A. Subsequently, a protein B engineered with a glycine residues at its N-terminus resolves the intermediate, thereby regenerating the active site cysteine on the mg SrtA and covalently conjugating protein A to the N-terminus of protein B (Fig. 1a)^34,35^. We first purified the HPV16 E6 peptide fused with H-2Kb-human IgG1 Fc to produce the mouse MHC-I‒E6‒IgG1 Fc fusion protein (Fig. 1b, c). This protein was then linked to a maleimide-LPET*G motif via thiol-maleimide reaction, where the asterisk indicates that the amide bond between the threonine and glycine residues was replaced with 2-hydroxyacetic acid via an ester linkage, generating mouse MHC-I‒E6‒IgG1 Fc-LPET*G. Additionally, erythrocyte membrane proteins were tagged with a glycine residue at their N-terminus. Through mg SrtA catalysis, we accomplished efficient surface conjugation of mouse MHC-I‒E6-IgG‒LPET*G to mouse erythrocytes (MHC-I‒E6‒mEry), yielding a remarkable 99.5% efficiency as confirmed by flow cytometry (Fig. 1d). Furthermore, the analysis of MHC‒I-E6‒mEry showed that there is no alteration in classical pro-phagocytic marker PS, anti-phagocytic marker CD47, cell morphology and deformability, suggesting the conjugation with the MHC-I complex does not compromise erythrocyte integrity (Supplementary Fig. S1a‒e)^36,37^. Consistently, when introduced into C57BL/6 mice, MHC-I‒E6‒mEry displayed a lifespan comparable to that of unmodified erythrocytes (Fig. 1e). Further investigation was conducted using in vitro assays to explore the potential of MHC-I‒E6‒mEry in activating antigen-specific T cells. The data revealed that MHC-I‒E6‒mEry triggered a significant activation of tumor-reactive T cells, with a threefold increase in IFN-γ-secreting T cells compared to controls when co-cultured with splenocytes from tumor-bearing mice (Fig. 1f). Moreover, these activated T cells triggered by MHC-I‒E6‒mEry exhibited heightened cytotoxicity against HPV16^+^ tumor cells, as validated by the in vitro killing assay (Fig. 1g). Also, we found that MHC-I‒E6‒mEry can effectively activate CD8^+^ T cells in the presence of splenocytes (Supplementary Fig. 2a‒c). In contrast, when CD8^+^ T cells were cultured alone with MHC-I‒E6‒mEry, such activation did not occur (Supplementary Fig. S2a‒c). This suggests a cooperative interaction where MHC-I‒Ery serves as a primary signal deliverer, while the requisite secondary signals, such as co-stimulatory molecules CD80/86, are provided by adjacent myeloid cells or others, thus prompting a complete T cell response^38^. Taken together, our results suggested that erythrocytes conjugated with MHC-I molecules alone can efficiently provide the first signal to T cells, which leads to their activation.

### MHC-I‒Ery specifically activates cytotoxic T cells from cancer patients

**Fig. 2:**
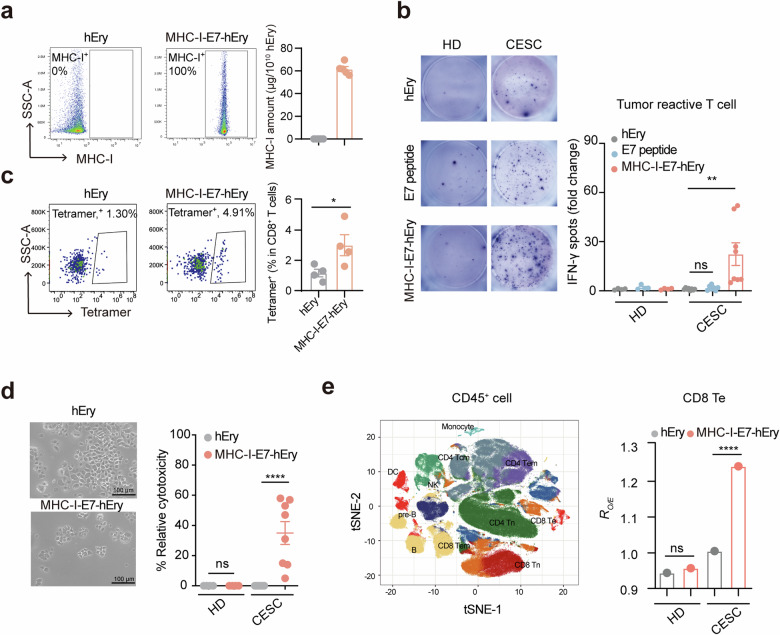
MHC-I‒E7‒hEry specifically activates cytotoxic CD8^+^ T cells from HPV16^+^ cervical cancer patients. **a** Left: flow cytometry plots of MHC-I‒E7‒hEry stained for human IgG. Right: quantification of MHC-I‒E7 proteins on erythrocytes analyzed by ELISA (*n* = 5 per group). **b** IFN-γ-secreting T cells were assessed using an ELISpot assay. PBMCs isolated from HPV16^+^ CESC patients or healthy donors (HD) were co-incubated with hEry, MHC-I‒E7‒hEry, or HPV16 E7 peptide (YMLDLQPET) for 48 h. Left: representative images of ELISpot wells. Right: the quantification of IFN-γ spots (CESC patients, *n* = 8; HD, *n* = 4). **c** Left: representative flow cytometry plots of HPV16-specific (tetramer^+^) CD8^+^ T cells after hEry or MHC-I‒E7‒hEry treatment. Right: HPV16-specific (tetramer^+^) CD8^+^ T cells from CESC patients after different treatments (*n* = 4 per group). **d** In vitro killing assay. PBMCs isolated from HPV16^+^ CESC patients or healthy donors were cultured with hEry or MHC-I‒E7‒hEry for 48 h. Following stimulation, CD8^+^ T cells were isolated and further co-cultured with Calcein AM-labeled Ca-ski tumor cells for additional 72 h. Left: representative images of tumor cells after the killing assay. Right: the relative cytotoxicity of CD8^+^ T cells against tumor cells under different treatments (CESC patients, *n* = 8; HD, *n* = 4). **e** Left: visualization of PBMCs from the CESC patient or the healthy donor after treatment with hEry or MHC-I‒E7‒hEry analyzed by mass cytometry using *t*-distributed stochastic neighbor embedding (*t*-SNE) plot. Right: proportion of effector CD8^+^ T cells in total T cells was estimated by *R*_*O/E*_ values. Data are presented as means ± SEM. Significance was determined by one-way ANOVA with Dunnett’s multiple-comparison test (**b**), unpaired *t*-test (**c**, **d**) or *chi*-square test (**e**). The significance levels are indicated as follows: ns, not significant; *P < 0.05; **P < 0.01; ****P < 0.0001.

We next explored the therapeutic potential of MHC-I‒E6/E7‒Ery in HPV16-associated malignancies. We developed human MHC-I‒E7‒Ery or MHC-I‒E7‒hEry by conjugating human erythrocytes with the HLA-A*02:01 proteins fused with the HPV16 E7 peptide YMLDLQPET (Supplementary Fig. S3)^32^. Flow cytometry analysis confirmed efficient conjugation, showing 100% of human erythrocytes with MHC-I molecules (Fig. 2a). ELISA tests showed approximately 61.7 μg of MHC-I molecules per 10^10^ erythrocytes, estimating 5 × 10^4^ MHC-I molecules on each erythrocyte (Fig. 2a). When co-cultured with PBMCs from HPV16^+^ cervical squamous cell carcinoma (CESC) patients, MHC-I‒E7‒hEry induced a notable 12.8-fold increase in IFN-γ-secreting T cells, which is in contrast with the effects of control groups and high-dose peptide treatments alone (Fig. 2b). This response was specific, as PBMCs from healthy donors did not exhibit increased cytotoxic response to MHC-I‒E7‒hEry, underscoring the targeted efficacy of this approach (Fig. 2b). Flow cytometry analysis further validated these findings, revealing an increased percentage of HPV16-specific (tetramer^+^) CD8^+^ T cells from CESC patients compared to healthy donors after stimulation with MHC-I‒E7‒hEry (Fig. 2c). Additionally, these CD8^+^ T cells displayed elevated expression levels of CD107a and 4-1BB upon exposure to MHC-I‒E7‒hEry, indicative of enhanced cytotoxic capability (Supplementary Fig. S4). Co-culture with Calcein AM-labeled Ca-ski tumor cells (HPV16^+^, HLA-A*02:01), derived from metastatic sites of cervical cancer patients, showed a significant reduction in fluorescent tumor cell counts compared to controls, confirming the killing potential of these CD8^+^ T cells activated by MHC-I‒E7‒hEry (Fig. 2d). Moreover, we also conducted mass cytometry analysis to further investigate the impact of MHC-I‒E7‒hEry on CD8^+^ T cells from CESC patients. Our results revealed an expansion of effector CD8^+^ T cells (CD45RA^+^CCR7^‒^) in the CESC patient following stimulation with MHC-I‒E7‒hEry, which was absent in samples from healthy donors (Fig. 2e; Supplementary Fig. S5). Taken together, these results provide strong evidence that MHC-I‒E7‒hEry can effectively incite the proliferation and enhance the cytotoxic function of tumor reactive CD8^+^ T cells in individuals with HPV16^+^ cervical cancer.

### MHC-I‒Ery exhibits strong anti-tumor activities and induces specific T cell activation in vivo

**Fig. 3:**
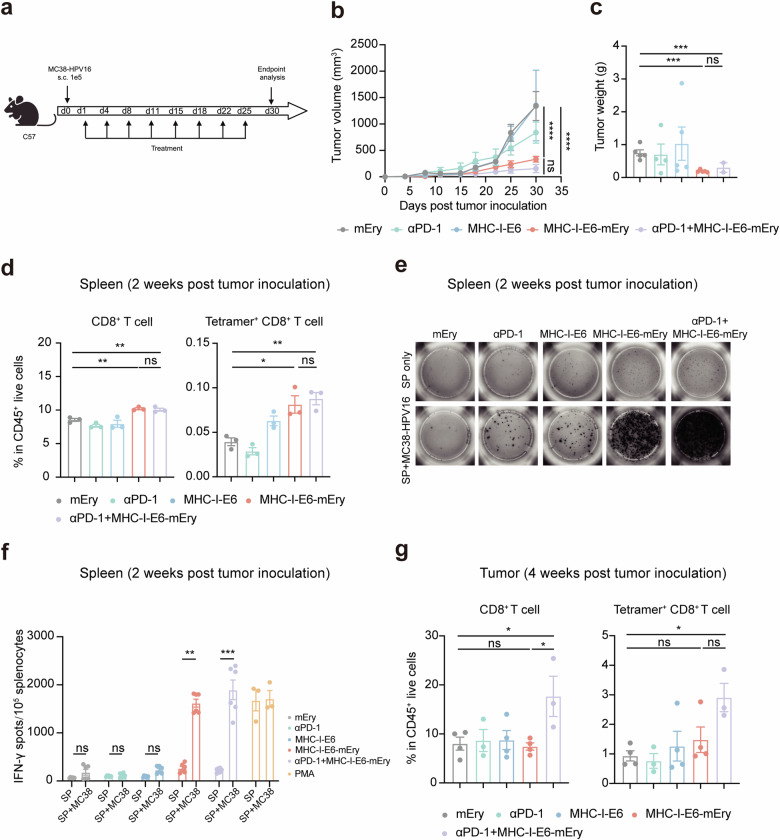
MHC-I‒E6‒mEry exhibits strong anti-tumor activities and induces specific T cell activation in vivo. **a** In vivo treatment regimen: C57BL/6 mice were subcutaneously injected with 1 × 10^5^ MC38-HPV16 tumor cells and received treatments of either mEry, αPD-1, MHC-I‒E6, MHC-I‒E6‒mEry, or MHC-I‒E6‒mEry plus αPD-1 twice weekly the day after tumor inoculation. **b** Tumor growth curves for MC38-HPV16 tumor model in C57BL/6 mice (*n* = 5 mice per group). **c** Tumor weight for MC38-HPV16 tumor model in C57BL/6 mice at the experimental endpoint (*n* = 5 mice per group). **d**‒**f** Splenocytes were isolated 2 weeks after tumor inoculation (*n* = 3 mice per group). **d** Proportions of CD8^+^ T cells and HPV16-specific (tetramer^+^) CD8^+^ T cells in the spleen from different groups were analyzed by flow cytometry. **e** IFN-γ-secreting T cells were tested by ELISpot assay. Splenocytes were co-cultured with MC38-HPV16 tumor cells for 48 h (*n* = 3 mice per group and two replicate wells per mouse). **f** Quantification of IFN-γ spot numbers in **e**. **g** Proportions of CD8^+^ T cells and HPV16-specific (tetramer^+^) CD8^+^ T cells in tumors from different groups were analyzed by flow cytometry 4 weeks after tumor inoculation (mEry, *n* = 4; αPD-1, *n* = 3; MHC-I‒E6, *n* = 4; MHC-I‒E6‒mEry, *n* = 4; MHC-I‒E6‒mEry plus αPD-1, *n* = 5). Data are presented as means ± SEM. Significance was determined by two-way ANOVA with Dunnett’s multiple-comparison test (**b**) or one-way ANOVA with Dunnett’s multiple-comparison test (**c**, **d**, **g**) or unpaired *t*-test (**f**). The significance levels are indicated as follows: ns, not significant; *P < 0.05; **P < 0.01; ***P < 0.001; ****P < 0.0001.

Next, we assessed the anti-tumor activities of MHC-I‒E6‒mEry in MC38-HPV16 tumor models (Fig. 3a). Notably, treatment with MHC-I‒E6‒mEry led to significant inhibition of tumor growth, with a 71.6% decrease in tumor volume compared to control subjects (Fig. 3b, c; Supplementary Fig. S6a, b). In contrast, administering MHC-I‒E6 proteins alone failed to elicit a noticeable response at similar dosage levels (Fig. 3b, c; Supplementary Fig. S6a, b). Further analysis showed an augmented presence of CD8^+^ T cells within the spleens of MHC-I‒E6‒mEry-treated mice (Fig. 3d). This was substantiated by a 1.3-fold increase in the population of HPV16-specific (tetramer^+^) CD8^+^ T cells and a substantial 7.0-fold elevation in IFN-γ-secreting CD8^+^ T cells upon tumor cell stimulation, compared to the group receiving only the protein treatment (Fig. 3d‒f). An up-regulation of tetramer^+^ CD8^+^ T cells was also observed in the lymph nodes and the TME, signifying a systemic activation of anti-tumor immunity upon MHC-I‒E6‒mEry administration (Fig. 3g; Supplementary Fig. S6c, d).

Considering the up-regulated levels of HPV16-specific CD8^+^ T cells in the TME, we postulated that a combined approach integrating MHC-I‒E6‒mEry with PD-1/PD-L1 checkpoint blockade might enhance anti-tumor effects. Supporting our hypothesis, we observed that treatment with αPD-1 antibodies alone slightly suppressed tumor progression. However, our combined regimen markedly escalated tumor repression, ultimately leading to complete remission in 40% of the treated mice (Fig. 3b, c; Supplementary Fig. S6a, b). Additionally, we observed a pronounced infiltration of CD8^+^ T cells within the tumors following the combination therapy with MHC-I‒E6‒mEry plus αPD-1, without detecting an analogous increase in splenic CD8^+^ T cell numbers (Fig. 3d‒g). In conclusion, our findings revealed that MHC-I‒E6‒mEry efficiently inhibits the growth of HPV16-positive tumors via the activation of peripheral immune responses and may work synergistically with αPD-1 therapy to enhance anti-tumor activity across different physiological compartments.

Inflammatory cytokines, including interferon (IFN)-γ, interleukin (IL)-6, and tumor necrosis factor (TNF)-α, play a major role in the development of immune-related adverse events^39,40^. Our results also showed that the levels of serum IFN-γ, IL-6, and TNF-α were not altered after three doses of MHC-I‒E6‒mEry treatment in MC38-HPV16 tumor-bearing mice compared to PBS-treated mice (Supplementary Fig. S7a‒c). Additionally, we assessed whether MHC-I‒E6‒mEry treatment induced damage to the liver and spleen by evaluating the level of alanine aminotransferase (ALT) in the serum^41^. Our results indicated that there was no significant change in the level of ALT in MHC-I‒Ery-treated mice compared to control mice (Supplementary Fig. S7d). Therefore, the repeated administration of MHC-I‒Ery is safe and well-tolerated in tumor-bearing mice.

### MHC-I‒Ery systemically reduces suppressive myeloid cells in tumor-bearing mice

Increased accumulation of immunosuppressive myeloid cells in the TME contributes to resistance to immunotherapies^42,43^. Previous studies have shown that the spleen undergoes enhanced extramedullary hematopoiesis, acting as a reservoir for immunosuppressive myeloid cells like myeloid-derived suppressor cells (MDSCs), which are known for their suppressive effect on T cells^14^. These cells can not only be recruited to tumors to promote tumor growth, but also induce tolerance in local T cells within the spleen^15,44^. Conversely, activated T cells can trigger apoptosis in MDSCs, leading to restored T cell function and enhanced anti-tumor immunity^45^. Given the successful activation of T cells by MHC-I‒E6‒mEry in the spleen, we wanted to investigate the impact of MHC-I‒E6‒mEry treatment on MDSCs systemically.

**Fig. 4:**
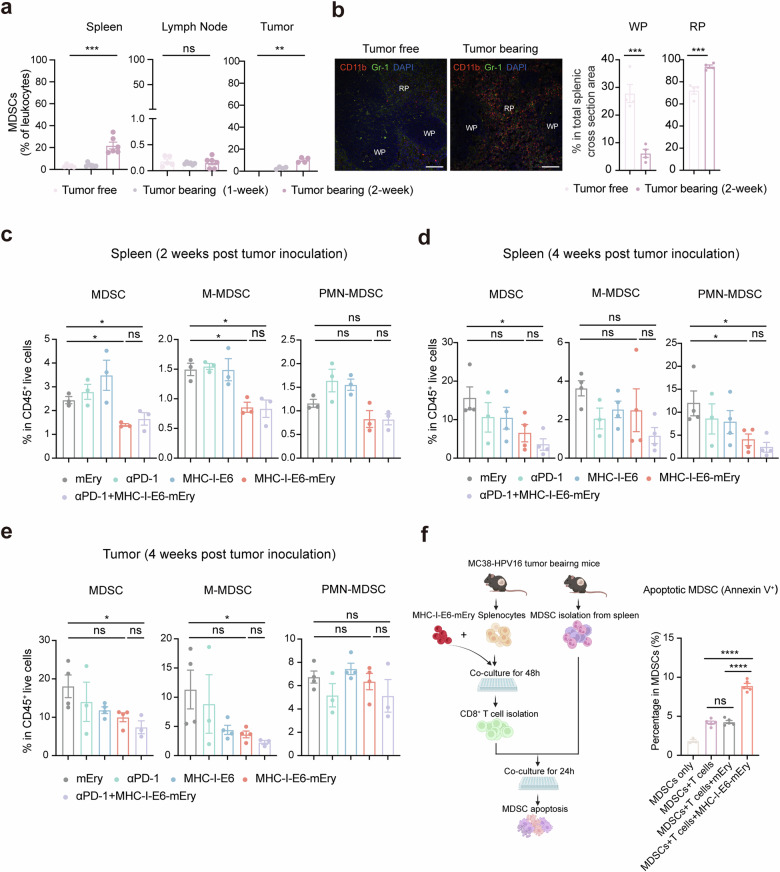
MHC-I‒E6‒mEry systemically reduces suppressive myeloid cells in tumor-bearing mice. **a** Proportions of MDSCs (CD11b^+^Gr-1^+^) were analyzed by flow cytometry in spleens, lymph nodes, and tumors during tumor growth over time (*n* = 6 mice per group). **b** Left: representative distribution patterns of MDSCs (CD11b^+^Gr-1^+^DAPI^+^) in the spleen from tumor-free or tumor-bearing mice (2 weeks after tumor inoculation) were assessed by immunofluorescence analysis. Scale bar: 100 μm. Right: proportions of WP and RP area in total splenic cross-section area of tumor-free or tumor-bearing mice (*n* = 4 mice per group). **c** Proportions of MDSCs and their subsets (M-MDSC, CD11b^+^Gr-1^+^Ly6C^hi^Ly6G^**‒**^; PMN-MDSC, CD11b^+^Gr-1^+^Ly6C^low^Ly6G^+^) in the spleen from different groups were analyzed by flow cytometry 2 weeks after tumor inoculation (*n* = 3 mice per group). **d** Proportions of MDSCs and their subsets in the spleen from different groups were analyzed by flow cytometry 4 weeks after tumor inoculation (mEry, *n* = 4; αPD-1, *n* = 3; MHC-I‒E6, *n* = 4; MHC-I‒E6‒mEry, *n* = 4; MHC-I‒E6‒mEry plus αPD-1, *n* = 5). **e** Proportions of MDSCs and their subsets in the tumor from different groups were analyzed by flow cytometry 4 weeks after tumor inoculation (mEry, *n* = 4; αPD-1, *n* = 3; MHC-I‒E6, *n* = 4; MHC-I‒E6‒mEry, *n* = 4; MHC-I‒E6‒mEry plus αPD-1, *n* = 5). **f** Schematic representation of in vitro induction of apoptotic MDSCs when co-cultured with CD8^+^ T cells activated by MHC-I‒E6‒mEry. Splenocytes from MC38-HPV16 tumor-bearing mice were isolated and co-cultured with mEry or MHC-I‒E6‒mEry for 48 h. CD8^+^ T cells were then enriched and co-cultured with splenic MDSCs isolated from tumor-bearing mice. The apoptotic MDSCs (Annexin V^+^) were analyzed by flow cytometry 24 h post co-culturing (*n* = 5 mice for each group, except for *n* = 3 mice for MDSC group). Data are presented as means ± SEM. Significance was determined by one-way ANOVA with Dunnett’s multiple-comparison test (**a**, **c**, **d**, **e**, **f**) or unpaired *t*-test (**b**). The significance levels are indicated as follows: ns, not significant; *P < 0.05; **P < 0.01; ***P < 0.001; ****P < 0.0001.

We first evaluated the expansion of MDSCs at different time points during tumorigenesis (Supplementary Fig. S8). Our flow cytometry analysis showed a subtle increase in MDSCs one week after MC38 tumor inoculation. However, a significant expansion of MDSCs was observed in the spleen and tumor two weeks after the inoculation, but not in the lymph nodes (Fig. 4a). Immunofluorescence analysis further validated these results, revealing a significant MDSC expansion in the red pulp (RP) of the spleen, accompanied by a 1.3-fold increase in the red marrow compartment (Fig. 4b). Conversely, the white pulp (WP), which is enriched for T cells and B cells and accounts for almost 30% of the spleen area in tumor-free mice, showed a 4.5-fold decrease in tumor-bearing mice due to the accumulation of suppressive myeloid cells, which is consistent with previous reports (Fig. 4b)^15^.

Next, we conducted a comprehensive analysis to evaluate changes in MDSCs in the spleen, lymph nodes, and tumors. Surprisingly, after MHC-I‒E6‒mEry treatment, a significant decrease in MDSC subsets, including both polymorphonuclear MDSCs (PMN-MDSCs) and mononuclear MDSCs (M-MDSCs), were observed in the spleen during the treatment, while the treatment with MHC-I‒E6 proteins or αPD-1 did not have the same effect (Fig. 4c, d). This phenomenon was also observed in the lymph nodes and tumors (Fig. 4e; Supplementary Fig. S6e). MHC-I‒E6‒mEry plus αPD-1 did not further decrease the percentage of MDSCs in either the spleen, lymph node, or tumor compared to the MHC-I‒E6‒mEry monotherapy (Fig. 4c‒e; Supplementary Fig. S6e).

To further understand the mechanism of MDSC reduction upon MHC-I‒E6‒mEry treatment, we examined the regulatory effects of MHC-I‒E6‒mEry on the interplay between CD8^+^ T cells and MDSCs in vitro. Our results revealed that CD8^+^ T cells stimulated by MHC-I‒E6‒mEry, but not mEry, triggered apoptosis of MDSCs (Fig. 4f). Additionally, we validated these findings in vivo through immunofluorescence analyses, where we observed the apoptosis of MDSCs in the spleen of mice treated with MHC-I‒E6‒mEry (Supplementary Fig. S9). Collectively, these findings further support the notion that MHC-I‒E6‒mEry treatment enhances the expansion of antigen-specific T cells and subsequently reduces the proportion of immunosuppressive cells such as MDSCs, thereby disrupting the function of the spleen as an MDSC reservoir involved in extramedullary hematopoiesis during tumor progression.

### MHC-I‒Ery demonstrates favorable safety profile in non-human primates

**Fig. 5:**
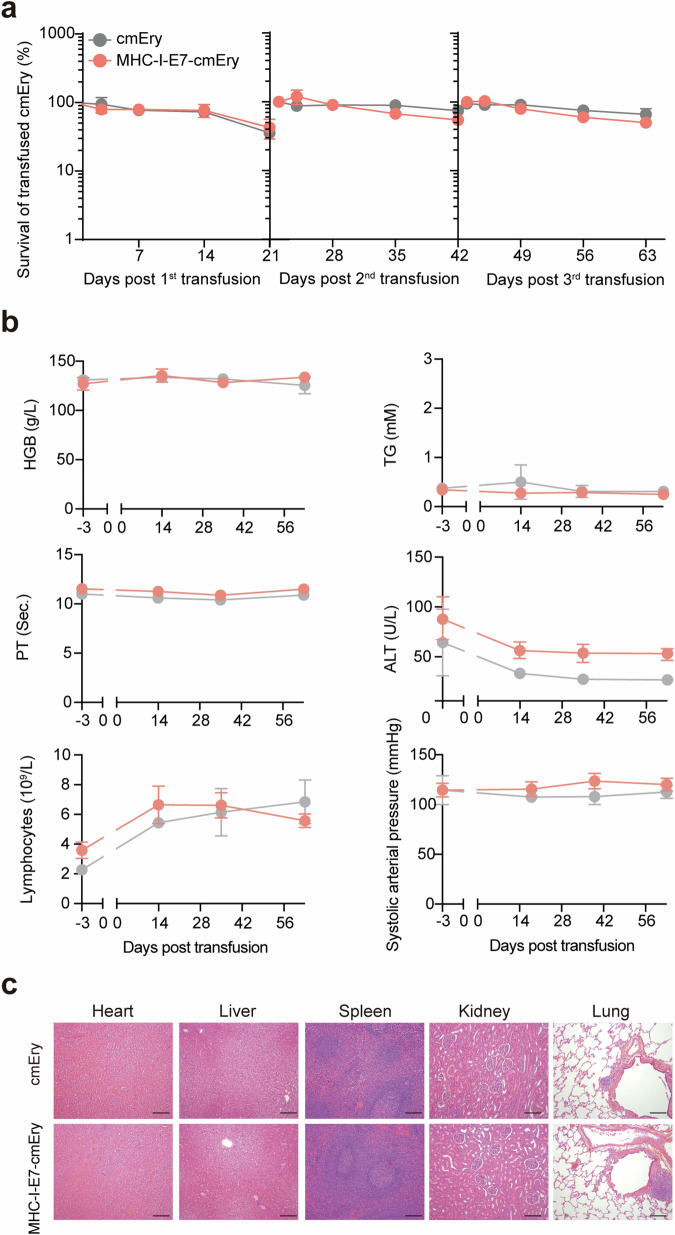
MHC-I‒E7‒cmEry demonstrates favorable safety profile in non-human primates. **a** Pharmacokinetic study of MHC-I‒E7‒cmEry in cynomolgus macaques. The monkeys were administered with Far-Red dye-labeled cmEry or MHC-I‒E7‒cmEry every 21 days. Blood was collected at specified time points (cmEry, *n* = 2; MHC-I‒E7‒cmEry, *n* = 4). **b** Safety assessments of MHC-I‒E7‒cmEry in cynomolgus macaques. Routine hematology (HGB, lymphocytes), coagulation tests (PT), serum biochemistry (TG, ALT), lymphocyte count and systolic arterial pressure were assessed throughout the experiment. **c** Representative images of H&E staining. A complete necropsy was performed on all animals. All organs were subjected to histopathological examination in each group using H&E staining. Scale bar: 20 μm. Data are presented as means ± SEM.

For further advancing MHC-I‒Ery into a potential therapy, we next evaluated the pharmacokinetic attributes and tolerability of MHC-I‒E7‒Ery in non-human primates. Six cynomolgus macaques (comprising an equal number of males and females) were enrolled in the study, with two receiving cynomolgus macaques’ erythrocytes (cmEry) and four treated with MHC-I‒E7‒cmEry. To mimic autologous transfusion in a clinical setting, blood samples were collected for preparing MHC-I‒E7‒cmEry. Prior to transfusion, MHC-I‒E7‒cmEry was labeled with Far-Red dye. MHC-I‒E7‒cmEry was administered every 21 days, and the survival rate was determined at specified time intervals. The results showed that both cmEry and MHC-I‒E7‒cmEry were well-tolerated, and no mortality occurred during the follow-up period. After three repeated doses, approximately 40% (average 43.3% and 36.0% respectively) of the transfused erythrocytes were detected in the peripheral blood at day 21, indicating comparable survival of both erythrocytes in cynomolgus macaques (Fig. 5a). Two macaques were monitored for an additional four-week recovery. No abnormal changes associated with MHC-I‒E7‒cmEry administration were observed during physical examinations or microscopic examinations conducted throughout the administration period and at the end of the recovery period. Basal physiological and toxicological indicators of each macaque were examined three days prior to the study and subsequently monitored at weeks 2, 5, and 9. The prothrombin time, which reflects the blood coagulation system, exhibited remarkable stability from the start to the end of the study (Fig. 5b). Additionally, hemoglobin and triglyceride levels remained unchanged, indicating that MHC-I‒E7‒cmEry autologous transfusion did not impair erythrocyte function or cause physiological discomfort (Fig. 5b). Alanine aminotransferase (ALT), an enzyme commonly monitored in erythrocyte-related transfusions as the liver and spleen are responsible for eliminating damaged erythrocytes, showed no significant changes, indicating that liver function was not affected by repeated MHC-I‒E7‒cmEry administration (Fig. 5b)^41,46^. Both groups exhibited a slight increase in lymphocyte count following blood collection and autologous transfusion; however, the absolute number remained within the normal range. The neutrophil-to-lymphocyte ratio (NLR) in MHC-I‒E7‒cmEry-treated animals also showed no significant change compared to cmEry-treated macaques, suggesting that the repeated transfusion of MHC-I‒E7‒cmEry did not induce unwanted inflammation in macaques (Supplementary Fig. S10)^47–49^. Systolic arterial pressure did not show significant fluctuations, indicating a safe profile among the tested macaques (Fig. 5b).

All cynomolgus macaques maintained stable weight, ingestion, heart rate, blood pressure, and body temperature (data not shown). Following autopsy, organ weight changes were measured to evaluate the toxicity^50^. Our results showed that there were no significant differences observed between the control and MHC-I‒E7‒cmEry groups (Supplementary Fig. S11). Additionally, histopathological examination using HE stains revealed normal morphology of the heart, liver, spleen, kidney, and lung in both groups (Fig. 5c). In summary, these results demonstrate that MHC-I‒E7‒cmEry is tolerated without discernible pathological sequelae or organ toxicity in non-human primate models, providing an important foundation for future clinical investigations of MHC-I‒E7‒Ery as a therapeutic option for HPV-associated malignancies.

## Discussion

In this study, we developed a therapeutic erythrocyte drug conjugated with MHC-I protein fused with HPV16 E6/E7 antigen peptide and demonstrated its robust capacity to activate antigen-specific CD8^+^ T cells and specifically enhance their cytotoxicity against tumor cells from HPV^+^ cancer patients in vitro. Additionally, MHC-I‒Ery exhibited potent anti-tumor activities in MC38-HPV16 tumor models. It successfully activated antigen-specific CD8^+^ T cells in the spleens and tumors. Moreover, MHC-I‒Ery treatment also induced a reduction in the reservoir of splenic MDSCs, leading to a decrease in MDSCs within the tumors. MHC-I‒Ery demonstrated excellent tolerability in non-human primates, and the pharmacokinetic study confirmed its favorable species migration properties.

Anti-cancer vaccines, involving the administration of specific proteins, peptides, or nucleic acids, have generated significant interest in cancer treatment. However, they have demonstrated limited reproducibility in inducing clinically meaningful regressions of established cancers^51,52^. These vaccines typically deliver antigens to local APCs in the lymph nodes, resulting in insufficient levels of antigen-specific CD8^+^ T cells in the circulation^53^. Although some patients have exhibited increased antigen-specific T cells in the circulation, tumor regression is often not achieved, potentially due to the immunosuppressive TME and the risk of selecting tumor cell variants that have lost HLA expression^54^. Efforts to enhance the efficacy of cancer vaccines, such as utilizing recombinant peptide‒MHC-I complexes or peptide‒MHC-I nanoparticle conjugations, have been made^55,56^. However, these medications can be rapidly cleared in the circulation, resulting in short half-lives^55^. Additionally, effective T cell activation requires multivalent T-cell receptor (TCR) cross-linking^57^. Monovalent engagement, whether with these fusion proteins or nanoparticle conjugations, often fails to induce sufficient signaling^58^. In contrast, MHC-I‒Ery presents an attractive solution. It boasts a long circulatory lifespan, enriched biodistribution to the spleen, high drug payload, and a flexible membrane, making it an ideal vehicle to overcome these limitations. Our findings also shed light on how erythrocytes can activate the immune system, potentially contributing to disease pathology. For example, MHC-positive erythrocytes might serve as immune activators, thereby facilitating disease progression^22^. Consequently, it is worth considering targeting these erythrocyte populations for the treatment of autoimmune diseases, such as systemic lupus erythematosus.

Precision in drug delivery is crucial for the success of cancer immune therapies. Various attempts involve utilizing different delivery strategies or injection routes to induce a more potent anti-tumor response in other lymphoid organs, such as the spleen^59^. However, the spleen contains a large number of phagocytes that rapidly clear drugs, thereby limiting the efficacy of transfused drugs^60^. For instance, some studies have delivered nanoparticle vaccinations via intravenous injection, aiming to activate the immune response in the spleen^53,61^. However, they only observe a short burst of antigen presentation in the spleen due to rapid nanoparticle clearance^59^. This can even lead to a lower amount of antigen-specific T cells compared to subcutaneous immunization to the lymph nodes. Additionally, the medications tend to accumulate predominantly in the liver and lung, potentially inducing unwanted toxicity and limiting efficacy^53^. In contrast, erythrocytes naturally pass through the spleen multiple times per day for pathogen filtration and clearance of aged or dysfunctional cells from circulation^11^. This frequent interaction between erythrocytes and immune cells in the spleen positions them as effective immune modulators. Pharmacokinetic data demonstrated that MHC-I‒Ery has a comparable lifespan in vivo to Ery both in mice and in non-human primates, suggesting that the enzyme-mediated engineering process did not induce further damage to the erythrocytes while achieving higher drug payload. Therefore, MHC-I‒Ery can maintain its natural characteristics and avoid rapid clearance by the MPS in the spleen. Consequently, MHC-I‒Ery can effectively activate immune cells in the spleen, which MHC-I protein or αPD-1 antibody cannot achieve. This feature distinguishes MHC-I‒Ery from other erythrocyte-engineering platforms that may induce erythrocyte damage, leading to the swift clearance of therapeutic erythrocyte drugs^23^.

Another obstacle to successful immunotherapy is the immunosuppression induced by tumors, which is caused by the accumulation of suppressive myeloid cells and regulatory T cells (Tregs) in the TME^62,63^. The substantial accumulation of immunosuppressive myeloid cells in tumor-bearing hosts plays a pivotal role in fostering resistance to immunotherapies^43^. Eliminating these cells within the TME has been shown to enhance T cell infiltration and restore responsiveness to immunotherapies^64,65^. Given that the spleen serves as a primary site for extramedullary hematopoiesis during tumorigenesis, it generates various myeloid suppressor cells, including erythroid progenitor cells (EPCs), PMN-MDSCs and M-MDSCs. The spleen acts as a reservoir for these immunosuppressive cells, contributing to the establishment of an immunosuppressive environment in the TME^66^. In this study, we showed that MHC-I‒Ery effectively reduces splenic MDSCs, potentially due to the activation of T cells by MHC-I‒Ery in the spleen^45^. Additionally, a concurrent decrease in MDSCs within the tumor sites and lymph nodes was also observed, providing clear evidence of the systemic activation of anti-tumor immunity by MHC-I‒Ery. The spleen’s tolerogenic role also relies on the enrichment of immunosuppressive cytokines^11^. For example, splenic macrophages can produce Interleukin 10 (IL-10), which converts antigen-specific CD4^+^ and CD8^+^ T cells into Tregs, essential for maintaining long-term tolerance^67^. Additionally, TGF-β released from myeloid cells inhibits the function of APCs, contributing to antigen tolerance within the spleen^68^. Our results showed that the expression of IL-10 and TGF-β1 was significantly reduced in myeloid cells especially MDSCs within spleens from MHC-I‒E6‒mEry-treated mice (Supplementary Fig. S12a, b). This suggests that MHC-I‒Ery can effectively reduce the production of immunosuppressive cytokines and overcome the local immunosuppressive microenvironment in the spleen, thereby further regulating the immunosuppression induced by tumors.

Overall, our study demonstrated the effectiveness of erythrocytes solely conjugated with MHC-I in activating the immune system. The research findings underscore the promising potential of MHC-I‒Ery as a comprehensive strategy for activating antigen-specific T cells and simultaneously diminishing suppressive myeloid cell populations. The demonstrated efficacy of erythrocytes conjugated with HPV antigen peptide-loaded MHC-I complexes in inhibiting tumor growth suggests that this approach could be extended to target a variety of tumor antigens. Furthermore, advancements in protein engineering may lead to the creation of peptide‒MHC-I protein complexes with improved T-cell receptor binding affinity, thereby potentially amplifying the activation effectiveness. Consequently, our studies propose an innovative method for manipulating the immune system to combat cancer.

## Materials and methods

### Animals

C57BL/6 mice, aged 6‒8 weeks, were sourced from the Laboratory Animal Resources Center at Westlake University. All mice were housed in specific pathogen-free (SPF) conditions, and all procedures involving animals strictly adhered to protocols approved by the Committee for Animal Research of Westlake University, following the guidelines outlined in the “Guide for the Care and Use of Laboratory Animals.” Cynomolgus macaques (Xishan Zhongke), aged 4 to 5 years and weighing between 3.5 to 4.0 kg at the initiation of dosing, were utilized in the study. The monkeys were accommodated in stainless-steel cages and were segregated during designated procedures. The animal room maintained a temperature range of 16 °C to 26 °C, with humidity levels set between 50% and 70%. The light cycle followed a 12-h light and 12-h dark pattern, except during specific procedures. The study protocols received approval from the Committee for Animal Research of Xishan Zhongke (IP20022717) and strictly adhered to the “Guide for the Care and Use of Laboratory Animals.”

### Human sample collection

Subject enrollment and the informed consent were approved by the Ethics Committee of Zhejiang Provincial People’s Hospital (2021KY036) for the collection of blood samples. The blood samples were then used for the preparation of MHC-I‒Ery and subsequent in vitro functional assays. Informed consent was obtained from all participants.

### Cell lines and cell culture

HEK293T (Co-bioer, CBP60440), MC38 (Co-bioer, CBPG0022) and Ca-ski (ATCC, CRM-CRL-1550) cells were obtained for the study. To introduce HPV16 E6/E7 genes into MC38, lentivirus was initially generated in HEK293T cells by transfecting them with the MSCV-HPV16 E6/E7 plasmid, and the resultant lentivirus was then applied to MC38 cells. Transduction efficiency was assessed via western blot using an HPV16 E6/E7 detection antibody (ThermoFisher, MA1-46057). MC38-HPV16 and Ca-ski tumor cell lines underwent regular authentication and mycoplasma contamination testing. Tumor cell lines were cultured in DMEM (Dulbecco’s Modified Eagle Medium, Sigma, D5921) supplemented with 10% FBS (Hyclone, SH30071.03HI) and 100 units/mL of penicillin and 100 μg/mL of streptomycin (Gibco, 15140122). All cell cultures were maintained at 37 °C in a humidified atmosphere with 5% CO_2_. Cell viability was evaluated using trypan blue staining.

### Protein expression and purification

To generate fusion proteins, we designed constructs for mouse MHC-I‒E6‒IgG1 Fc and human MHC-I‒E7‒IgG1 Fc. The mouse construct includes a structure of mouse β2M signal peptide-HPV16 E6 epitope (KCLKFYSKI)-GCGAS(G_4_S)-mouse β2M-(G_4_S)_4_-mouse H-2Kb-(G_4_S)-human IgG1 Fc. The human construct comprises a structure of human β2M signal peptide-HPV16 E7 epitope (YMLDLQPET)-GCGAS(G_4_S)-human β2M-(G_4_S)_4_-HLA-A*02:01-G_4_S-human IgG1 Fc. The cDNA and electroporation buffer were mixed and transferred to an electroporation cuvette. Electroporation into CHO cells were performed following the manufacturer’s protocols (Ettabiotech), optimized for CHO cells. After 7 days, supernatants were collected by centrifugation at 14,000× *g* for 40 min at 4 °C and filtered through a 0.22 μm filter. The fusion proteins were substantially enriched using protein A chromatography, followed by concentration with an Amicon Ultra-15 Centrifugal Filter Unit (Millipore, UFC9050). The concentrated protein was loaded onto a Chromdex 200 PG column (Bestchrom, AG200802) equilibrated with PBS, and target protein fractions were collected. The protein was concentrated and stored at ‒80 °C. For SDS-PAGE analysis, purified proteins were diluted in reducing (Beyotime, P0015) or non-reducing sample buffer (Beyotime, P0016N) before electrophoresis. Both reduced and non-reduced samples were run on the same gels to enable on-gel comparison of the migration of the reduced/non-reduced parent protein. Prior to gel application, all samples were heated in a boiling water bath for 5 min. Prepared solutions (10 μL) were applied to SurePAGE™ (genscript, M00657) for reducing conditions and non-reducing conditions. Molecular weight markers (TransGen, DR401) were run concurrently on all gels for molecular weight determination.

### Generation of MHC-I‒Ery

Erythrocytes were separated from peripheral blood from humans, cynomolgus macaques or mice using density gradient centrifugation and washed three times with PBS. For erythrocyte treatment, they were first reacted with 2.5 mM TCEP (Sigma, 646547) at room temperature (RT) for 1 h, followed by three washes with PBS. GAASK-6-maleimide (Beijing Siruibio Technology) at a final concentration of 100 μM was dissolved in phosphate buffer at 37 °C. Subsequently, 1 × 10^9^ erythrocytes were incubated with 50 μM GAASK-6-maleimide at 37 °C for 30 min and washed three times with PBS. For protein modification, mouse MHC-I‒E6‒IgG1 Fc or human MHC-I‒E7‒IgG1 Fc fusion protein was conjugated to the maleimide-LPET*G motif through a thiol-maleimide reaction, resulting in the formation of MHC-I‒IgG Fc-LPET*G. Using the catalytic effect of mg SrtA, MHC-I‒IgG Fc-LPET*G was successfully conjugated to the surface of erythrocytes, washed with PBS three times to yield the final product. Following the reaction, the labeling efficiency of erythrocytes was assessed through flow cytometry using anti-human IgG-PE (ThermoFisher, MA1-10377).

### PS and CD47 analysis

To detect PS exposure, MHC-I‒E6‒mEry or mEry was incubated with Annexin V (Biolegend, 640906) at RT for 20 min. To quantify CD47 expression, MHC-I‒E6‒mEry or mEry was stained with rat anti-CD47 monoclonal antibody (Biolegend, 127507) at 4 °C for 30 min. Afterward, cells were washed twice with PBS and resuspended in FACS buffer before analysis. PS exposure and CD47 expression were assessed by flow cytometry and analyzed with FlowJo software. The data shown are representative of two independent experiments.

### Imaging flow cytometry

MHC-I‒E6‒mEry and mEry were stained with anti-human IgG antibody (Abcam, ab6854) and anti-Ter119 antibody (BD Biosciences, 557909), and incubated for 15 min at RT. Cells were then washed twice with PBS and analyzed by imaging cytometry (Merk, FC-ISX). Images were acquired using the ImageStreamX mark II. At least 2 × 10^5^ cells were collected from each sample. Images were analyzed using IDEAS 6.3 software (Amnis, Part of Luminex, Austin, TX). The data shown are representative of two independent experiments.

### Benzidine and Giemsa staining

MHC-I‒E6‒mEry and mEry were subjected to Benzidine and May-Grunwald Giemsa stainings by standard procedures. Briefly, erythrocytes were first conducted benzidine staining. After spinning and drying, the MHC-I‒E6‒mEry and mEry were fixed in methanol solution for 5 min. Then, the DAB working solution containing 0.03% hydrogen peroxide (Sigma-Aldrich, D5905) was added and allowed to incubate at RT for 1 h. After that, the samples were rinsed twice with MQ water and left to dry at RT for 2 h. Next, we performed the Giemsa staining using the Rapid Giemsa Stain Kit (Beyotime, E607314). The working solution was prepared by mixing Solution I (component A) and Solution II (component B) at a 1:9 ratio followed by thorough mixing. The slides were immersed in the Giemsa staining working solution and incubated at RT for 30 min. After being rinsed twice with MQ water and air-dried at RT, the samples were captured using a Nikon fully automatic fluorescence upright microscope. The data shown are representative of two independent experiments.

### Osmotic fragility assay

The osmotic fragility of erythrocytes was determined using the Erythrocyte Incubation Penetration Fragility Detection Kit (YaJi Biotechnology J, YC0227) First, different concentrations of sodium chloride were prepared. Then, MHC-I‒E6‒mEry and mEry were added to the different concentrations of NaCl and mixed well, followed by incubation at RT for 20 min. After centrifugation, the supernatant was taken and placed in a 96-well plate. The absorbance at a wavelength of 540 nm was measured using a multi-function microplate reader, Thermo Varioskan LUX. NaCl phosphate buffer was used as a blank tube for zero calibration. The hemolysis percentage was calculated as follows: using 17.1 mM NaCl as the absorbance of the fully hemolyzed tube set to 100%, the hemolysis percentage of the corresponding NaCl concentration was calculated. The calculation formula is Hemolysis Percentage (%) = (Absorbance of each well ‒ Blank tube reading)/Absorbance of fully hemolyzed tube × 100%. The data shown are representative of two independent experiments.

### The determination of drug payload of MHC-I‒E7‒hEry

Erythrocytes were lysed using the RIPA buffer (Cell Signaling Technology, 9806). Flatbottomed 96 well plates were coated with 1 μg/mL of anti-HPV E7 peptide (YMLDLQPET)-GCGAS(G_4_S) Ab (Leadgene Biomedical) in 100 μL of PBS at 4 °C overnight. Three times washing was performed between each step using PBS with 0.1% Tween. All incubations were maintained for 1 h at 37 °C unless noted otherwise. Coated plates were blocked by 5% skim milk solution. After blocking, erythrocyte lysates were added to the plates, incubated, and washed. Goat anti-Human IgG-Fc (HRP) (ThermoFisher, A18817) and tetramethylbenzidine substrate solution (Solarbio, PR1210) were subsequently added for detection. The absorbance at 450 nm was measured using Molecular Devices’ iD5 luminometer. The data shown are representative of three independent experiments.

### Pharmacokinetic study of MHC-I‒E6‒mEry and MHC-I‒E7‒cmEry

MHC-I‒E6‒mEry or MHC-I‒E7‒cmEry were generated following the methods described above. Subsequently, erythrocytes were suspended in PBS at a concentration of 5 × 10^9^/mL and labeled with 1 μM Far-Red dye (ThermoFisher, C34564). The cells were incubated in the dark at 37 °C for 20 min, and the reaction was halted by adding PBS containing at least 1% BSA. After an additional 5 min of incubation, the cells underwent two washes with PBS. Far-Red dye-labeled erythrocytes were finally suspended in PBS and administered intravenously to mice or monkeys. The proportion of transfused erythrocytes circulating at various time points post-transfusion was measured. At defined intervals post-transfusion, a blood sample (5 μL for mice, 2 mL for monkeys) was obtained for flow cytometric detection of fluorescently labeled erythrocytes. The samples were analyzed using flow cytometry. Erythrocyte survival was determined by calculating the ratio of the percentage of Far-Red^+^ erythrocytes at indicated time points divided by the percentage of Far-Red^+^ erythrocytes at the first time point (10 min for mice, 30 min for monkeys) after injection. The data were normalized to the fraction at the first time point after injection, set as 100%. The pharmacokinetic study of MHC-I‒E6‒mEry in mice was conducted three times.

### IFN-γ ELISpot assay

The IFN-γ ELISpot Kit (Mabtech, 3420-2AST-2 for human, 3321-4AST-2 for mouse) was employed following the manufacturer’s instructions. For the ex vivo functional assessment of MHC-I‒E6‒mEry, splenocytes (2 × 10^5^ cells per well) from MC38-HPV16 tumor-bearing mice were incubated with 1 × 10^7^ MHC-I‒E6‒mEry, mEry, MHC-I‒E6**-**low (0.5 μg/mL), MHC-I‒E6**-**high (25 μg/mL), or HPV16 E6 peptide (5 μg/mL) in RPMI-1640 supplemented with 10% FBS for 48 h in a 96-well plate. A concentration of 1 μg/mL of PMA was added for the positive control. In addition, CD8^+^ T cells (2 × 10^5^ cells per well, high; 2 × 10^4^ cells per well, low) from the spleen of MC38-HPV16 tumor-bearing mice were co-incubated with either 1 × 10^7^ mEry or MHC-I‒E6‒mEry in RPMI-1640 supplemented with 10% FBS for 48 h in a 96-well plate. A concentration of 1 μg/mL of PMA was added for the positive control. For the ex vivo functional evaluation of MHC-I‒E7‒hEry, PBMCs isolated from HPV16^+^ patients were plated in duplicate at a density of 2 × 10^5^ cells per well and incubated with 1 × 10^7^ MHC-I‒E7‒hEry or hEry in RPMI-1640 supplemented with 10% FBS. HPV16 E7 peptides were added to ELISpot wells at 20 μg/mL and incubated for 48 h at 37 °C. Secreted IFNγ was detected by biotinylated anti-IFNγ mAb, followed by reaction with streptavidin-HRP and colorimetric substrate BCIP/NBT. The number of specific IFNγ-secreting T cells was then calculated using an automated spot counter (ImmunoSpot® CTL S6 Micro Analyzer; Cellular Technology Limited) and analyzed as spot forming units (SFU) per well. Raw data included images for each well for visual quality checking and the corresponding SFU counts per well. All data shown are representative of at least two independent experiments.

### MDSC and CD8^+^ T cell isolation

For the isolation of MDSCs (CD11b^+^Gr1^+^), splenocytes from mice bearing MC38-HPV16 tumors (tumor volume ≈ 1500 mm^3^) were subjected to magnetic isolation using the EasySep™ Mouse MDSC (CD11b^+^Gr1^+^) Isolation Kit (Stem Cell Technologies, Cat# 19867). For the isolation of CD8^+^ T cells, splenocytes from mice bearing MC38-HPV16 tumors (tumor volume ≈ 200 mm^3^) were subjected to magnetic isolation using the EasySep™ Mouse Naïve CD8^+^ T Cell Isolation Kit (Stem Cell Technologies, Cat# 19853). Single-cell suspensions of the spleen were obtained by passing the tissue through a 70-μm cell strainer (Falcon, 352350), followed by erythrocyte lysis (BD, 555899). To remove non-MDSCs or non-CD8^+^ T cells, biotinylated antibodies and streptavidin-coated magnetic particles (RapidSpheres™) were employed. The labeled cells were then separated using an EasySep™ magnet, resulting in the isolation of MDSCs and CD8^+^ T cells suitable for downstream applications.

### In vitro killing assay

For the ex vivo functional assessment of MHC-I‒E6‒mEry, splenocytes from MC38-HPV16 tumor-bearing mice were incubated with MHC-I‒E6‒mEry (splenocytes: MHC-I‒E6‒mEry = 1:50) for 48 h at 37 °C. After stimulation, the splenocytes were enriched for CD8^+^ T cells using the CD8^+^ T Cell isolation kit (Stem cell, 19853). Isolated CD8^+^ T cells were co-cultured with Calcein AM (Thermo Fisher, C3099) stained target MC38-HPV16 tumor cells for 72 h (effector cells: target cells = 100:1). The fluorescence signals of tumor cells were measured using Molecular Devices’ iD5 luminometer. The cytotoxic potential of the isolated CD8^+^ T cells was calculated by the reduction in fluorescence signals of tumor cell counts compared to controls. For the ex vivo functional assessment of MHC-I‒E7‒hEry, PBMCs from HPV16^+^ patients or healthy donors were incubated with MHC-I‒E7‒hEry (PBMCs:MHC-I‒E7‒hEry = 1:50) for 48 h at 37 °C. After stimulation, the patient PBMCs were enriched for CD8^+^ T cells using the CD8^+^ T Cell isolation kit (Stem Cell, 17953). Isolated CD8^+^ T cells were co-cultured with Calcein AM stained target Ca-Ski tumor cells obtained from ATCC (CRL-1550TM) for 72 h (effector cells:target cells = 100:1). The fluorescence signals of tumor cells were measured using Molecular Devices’ iD5 luminometer. The cytotoxic potential of the isolated CD8^+^ T cells was calculated by the reduction in fluorescence signals of tumor cell counts compared to controls. The expression of 4-1BB (Biolegend, 309814), CD107a (Biolegend, 328620), and fluorochrome-conjugated MHC tetramer (MBL, TB-0173-1) on CD8^+^ T cell surface was also assessed by flow cytometry. The data shown are representative of at least three independent experiments.

### In vitro co-culture of MDSCs and CD8^+^ T cells

C57BL6/J mice were subcutaneously inoculated with 1 × 10^5^ MC38-HPV16 tumor cells. When the tumors reached a volume of approximately 200 mm^3^, the spleen was isolated and prepared into a single cell suspension which was then co-cultured with mEry or MHC-I‒E6‒mEry at a ratio of 1:50 (2 × 10^5^ splenocytes and 1 × 10^7^ mEry or MHC-I‒E6‒mEry) for 48 h. Subsequently, CD8^+^ T cells were isolated by magnetic sorting. Meanwhile, MDSCs were isolated from the spleen of MC38-HPV16-tumor bearing mice when the tumors reached a volume of approximately 1500 mm^3^ by magnetic sorting. Following that, CD8^+^ T cells and MDSCs were co-cultured at a 1:1 (2 × 10^4^ CD8^+^ T cells and 2 × 10^4^ MDSCs) for 24 h, and the apoptosis of MDSCs was assessed by flow cytometry. Apoptotic MDSCs were identified with Annexin V^+^ (Biolegend, 640906) populations. The data shown are representative of two independent experiments.

### In vivo mouse models

C57BL6/J mice were subcutaneously inoculated with 1 × 10^5^ MC38-HPV16 tumor cells on Day 0 and were subsequently randomly assigned to five experimental groups. The treatment groups included administration of mEry (1 × 10^9^ cells, i.v.), MHC-I‒E6‒mEry (1 × 10^9^ cells, i.v.), αPD-1 (BioXcell, RMP1-14; 0.5 mg/kg, intraperitoneally (i.p.)), MHC-I‒E6 (0.5 mg/kg, i.v.), and a combination of MHC-I‒E6‒mEry (1 × 10^9^ cells, i.v.) plus αPD-1 (0.5 mg/kg, i.p.) twice a week starting from the day after tumor inoculation. Body weight and tumor volume were monitored every three days throughout the treatment period. After either 2 weeks (for early-stage analysis, *n* = 3 mice/group) or 4 weeks (for late-stage analysis, *n* = 3‒5 mice/group) of treatment, animals were euthanized, and the spleens, lymph nodes, and tumors were collected. Spleens and lymph nodes were dissociated into single-cell suspensions, and tumors were processed using the Tumor Dissociation Kit (Miltenyi Biotec, 130-096-730). Immune cell subsets were identified using specific antibodies: anti-CD8α antibody (Thermo Fisher, A15386), antigen-specific tetramer (MBL, TB-7400-K1), anti-mouse CD45 antibody (Biolegend, 103114), anti-mouse/human CD11b antibody (Biolegend, 101263), anti-mouse Ly-6C antibody (Biolegend, 128032), and anti-mouse Ly6G antibody (Biolegend, 127624). All data shown are representative of at least three independent experiments.

### Preclinical safety evaluation in murine tumor models

C57BL/6 mice were subcutaneously injected with 1 × 10^5^ MC38-HPV16 tumor cells. On the second day after tumor inoculation, the mice were treated with PBS, mEry, or MHC-I‒E6‒mEry i.v. twice a week. On day 11, serum samples were collected for analysis. For the LPS positive control group, mice were i.p. injected with 5 mg/kg LPS and serum samples were collected 6 h later. The concentrations of IFN-γ (Absin, abs520007), IL-6 (Proteintech, KE10007), and TNF-α (Proteintech, KE10007) in serum were quantitatively analyzed by ELISA assay. Simultaneously, serum ALT levels were also monitored (Solarbio, BC1555). The data shown are representative of two independent experiments.

### Immunofluorescence analysis

For the analysis of MDSC expansion in tumor progression, C57BL/6 mice were subcutaneously inoculated with 1 × 10^5^ MC38 tumor cells per mouse. Spleens were harvested for MDSC immunofluorescence analysis 2 weeks after tumor inoculation. Spleens from tumor-bearing mice, as well as additional tumor-free mice, were isolated for analysis. The division between the WP and RP areas in the spleen was established based on their specific characteristics, using markers CD11b, Gr-1, and DAPI. The RP is mainly filled with erythrocytes and myeloid cells, while the WP is composed primarily of lymphocytes with a higher density of nucleated cells^69^. The areas of these two regions were then quantified using ImageJ software. For the analysis of MHC-I‒E6‒mEry induced MDSC apoptosis in vivo, MC38-HPV16 tumor-bearing mice (tumor volume of approximately 200 mm^3^) were injected with MHC-I‒E6‒mEry and after 2 h, spleens were isolated for immunofluorescence staining. Tissues were fixed in 4% paraformaldehyde (PFA) at 4 °C for 4‒5 h, followed by incubation in 30% sucrose at 4 °C for 12‒16 h until tissue sinking occurred. Subsequently, the tissues were embedded in Tissue-Tek OCT compound (Sakura, Torrance, USA). Cross sections of 10 μm thickness were cut using a CM1950 cryostat (Leica, Wetzlar, Germany). The sections were fixed in 4% PFA and blocked with 10% horse serum. An overnight incubation in PBS at 4 °C was performed, followed by incubation with anti-CD11b antibody (Abcam, ab128797, 1:100), anti-Ly-6G/Ly-6C antibody (Thermo Fisher, 14-5931-85, 1:100), anti-CD8 antibody (Thermo Fisher, 42-0081-82, 1:100), anti-Cleaved Caspase-3 antibody (CST, #9661, 1:100) in PBS overnight. Subsequently, the sections were washed three times for 5 min each with PBST. Fluorescently conjugated secondary antibodies (1:500 dilution in PBS) were then incubated at RT for 1 h. The secondary antibodies used were Donkey Anti-Rat IgG H&L (Alexa Fluor® 555) (Abcam, ab150154, 1:500), Donkey anti-Rabbit IgG (H + L) (Alexa Fluor® 488) (Thermo Fisher, A-21206, 1:500), Donkey anti-Rabbit IgG H&L (Alexa Fluor® 647) (Abcam, ab150075, 1:500). Imaging was performed using a confocal FV3000 microscope (Olympus FV3000 upright, FV3000-BX63), and analysis was conducted using Olympus FV31S-SW software. All data shown are representative of at least two independent experiments.

### Real-time PCR for cytokine expression analysis

C57BL/6 mice were subcutaneously injected with 1 × 10^5^ MC38-HPV16 tumor cells. The mice then received intravenous injections of mEry or MHC-I‒E6‒mEry twice weekly, starting one day after tumor inoculation. On day 7 post-inoculation, splenocytes were isolated and subjected to flow cytometry to isolate the population of MDSCs, while excluding other myeloid cell populations. RNA was extracted from the isolated cells using the FastPure Cell/Tissue Total RNA Isolation Kit (Vazyme, RC101). The RNA was then reverse transcribed into cDNA using the PrimeScript™ RT Reagent Kit with gDNA Eraser (Perfect Real Time) (Takara, RR047B). Quantitative polymerase chain reaction (qPCR) was performed using the TB Green Premix Ex Taq (Takara, RR420B) on a Lightcycler 2000 instrument (Roche, #CLB20405). The primer sequences used for amplifying the target genes were as follows: *IL-10* (Forward: GCTCTTACTGACTGGCATGAG; Reverse: CGCAGCTCTAGGAGCATGTG), *TGF-β1* (Forward: CTCCCGTGGCTTCTAGTGC; Reverse: GCCTTAGTTTGGACAGGATCTG), and GAPDH (Forward: AGGTCGGTGTGAACGGATTTG; Reverse: TGTAGACCATGTAGTTGAGGTCA). The obtained qPCR data were normalized to GAPDH expression levels using the ΔΔCt method. The data were further expressed as fold change relative to the control group (2^‒^^Δ^^Δ^^Ct^). The data shown are representative of two independent experiments.

### Preclinical safety evaluation in non-human primates

Six cynomolgus macaques (50% male and 50% female) were purchased from Suzhou Xishan Zhongke Laboratory Animal Co., Ltd. All monkeys were maintained in specific pathogen-free rooms, temperature 18.0 –25.4 °C, humidity of 40.3%–86.1%, 12-h light/dark cycle. Water and standard laboratory fodder/chow were allowed ad libitum. Cynomolgus macaques were randomly assigned to two groups (*n* = 2 in control group and *n* = 4 in MHC-I‒E7‒cmEry treatment group, male:female = 1:1 in each group). 15 mL peripheral blood of each cynomolgus macaque were collected to prepare cmEry or MHC-I‒E7‒cmEry. Three days post blood collection, cmEry or MHC-I‒E7‒cmEry were autologously transfused to donor macaque. The treatment was administrated 3 times for total 9 weeks. Two animals were allowed to recovery for 4 weeks after the last administration. The regular clinical manifestation evaluation included body weight, food consumption, body temperature, respiratory rhythm, blood pressure, electrocardiogram, hematology, hepatic function and immune markers. The cmEry group and two of MHC-I‒E7‒cmEry-treated macaques were subjected to necropsy after treatment administration, the rest two macaques of MHC-I‒E7‒cmEry-treated group were sacrificed after the 4-week recovery. Major organs were isolated, weighed, and fixed in a 10% formaldehyde solution. The organ coefficients (organ/body weight ratios) were calculated as the ratio of specific organ weight to body weight at the endpoint. The organs were processed and embedded in paraffin for sectioning. The sections, cut to 4-µm thickness, were stained with hematoxylin and eosin for histopathological examination.

### Flow cytometry

Mice were euthanized to assess immune parameters in spleens, lymph nodes and tumors. Splenic cell and lymph node suspensions were prepared by pressing the tissue through 70-μm cell strainers, followed by erythrocyte lysis. Tumors were cut into small pieces and digested using the Tumor Dissociation Kit (Miltenyi Biotec, 130-096-730). Single-cell suspensions from tumors were filtered through 70-μm cell strainers and then subjected to red blood cell lysis. The samples were resuspended in flow cytometry staining buffer (eBioscience™, 00-4222-57) and viability staining was used to exclude dead cells. The samples were then labeled with the specified antibodies for 20 min at RT. Flow cytometry analyses were conducted on a CytoFLEX LX-5L Flow Cytometer (Beckman Coulter), and the data were analyzed using FlowJo software (v.10.4).

### Mass cytometry

PBMCs from healthy donors or CESC patients were collected for mass cytometry analysis after incubation with hEry or MHC-I‒hEry for 72 h. For sample preparation, cells were washed with 1× PBS and then stained with 100 μL of 250 nM cisplatin (Fluidigm) for 5 min on ice to exclude dead cells, and then incubated in Fc receptor blocking solution before stained with surface antibody cocktail for 30 min on ice. Cells were washed twice with FACS buffer (1 × PBS + 0.5% BSA) and fixed in 200 μL of intercalation solution (Maxpar Fix and Perm Buffer containing 250 nM 191/193Ir, Fluidigm) overnight. After fixation, cells were washed once with FACS buffer and then perm buffer (eBioscience, 88-8824-00), stained with intracellular antibody cocktail for 30 min on ice. After fixation, cells were washed once with FACS buffer and then with perm buffer (eBioscience, 88-8824-00). Subsequently, the cells were stained with a cocktail of intracellular antibodies for 30 min on ice. After being washed, the cells were resuspended in deionized water and mixed with 20% EQ beads (Fluidigm). The data were acquired on a mass cytometer (Helios, Fluidigm). For data analysis, manually gated four PBMC samples were downloaded as FCS files from CellEngine, and CATALYST R/Bioconductor package was used to import FCS files into R (Rahim et al., 2023a). The FlowSOM clustering algorithm, available through the CATALYST was used to generate 40 clusters based on cell specific markers (CD45, CD45RA, CD45RO, CD3, CD4, CD8a, CD197/CCR7, CD183/CXCR3, CD127/IL-7Ra, CD25/IL-2 R, CD152/CTLA-4, CD278/ICOS, CD85j, CD123/IL-3 R, CD279/PD-1, CD28, CD39, CD27, CD194/CCR4, TCRgd, CD33, CD20, CD19, CD38, CD185/CXCR5, CD196/CCR6, CD24, CD57, CD56, CD94, CD16, CD161, CD11b, CD14, CD11c, HLA-DR, CD86.2, Gd156Di, CD274/PD-L1, CD66b, IgD). Clusters were visualized using plotDR using tSNE mode and heatmaps via the plotExprHeatmap, both available through CATALYST. We use the ratio of observed cell numbers to random expectation calculated by chi-square test (*R*_*O/E*_) in each cluster, which was used to adjust cell-sampling biases for each sample.

### Statistical analysis

All experiments were repeated at least twice. Statistical analysis was conducted using Prism 7.0 (GraphPad). The number of animals is specified in each figure legend. Data are presented as means ± SEM. Statistical analyses used unpaired *t*-test, and one-way or two-way ANOVA. Statistical significance is indicated as follows: ns, not significant; *P < 0.05; **P < 0.01; ***P < 0.001; ****P < 0.0001.

## Supplementary information

Supplementary figures
