# Supplementary material for: Developing an erythrocyte‒MHC-I conjugate for cancer treatment
Source: Cell Discov. 2024 Oct 1;10:99. doi: 10.1038/s41421-024-00713-9 (PMC11443136; doi:10.1038/s41421-024-00713-9)
Supplement: Supplementary file 1 — Supplementary figures [file 41421_2024_713_MOESM1_ESM.pdf]

Fig. S1

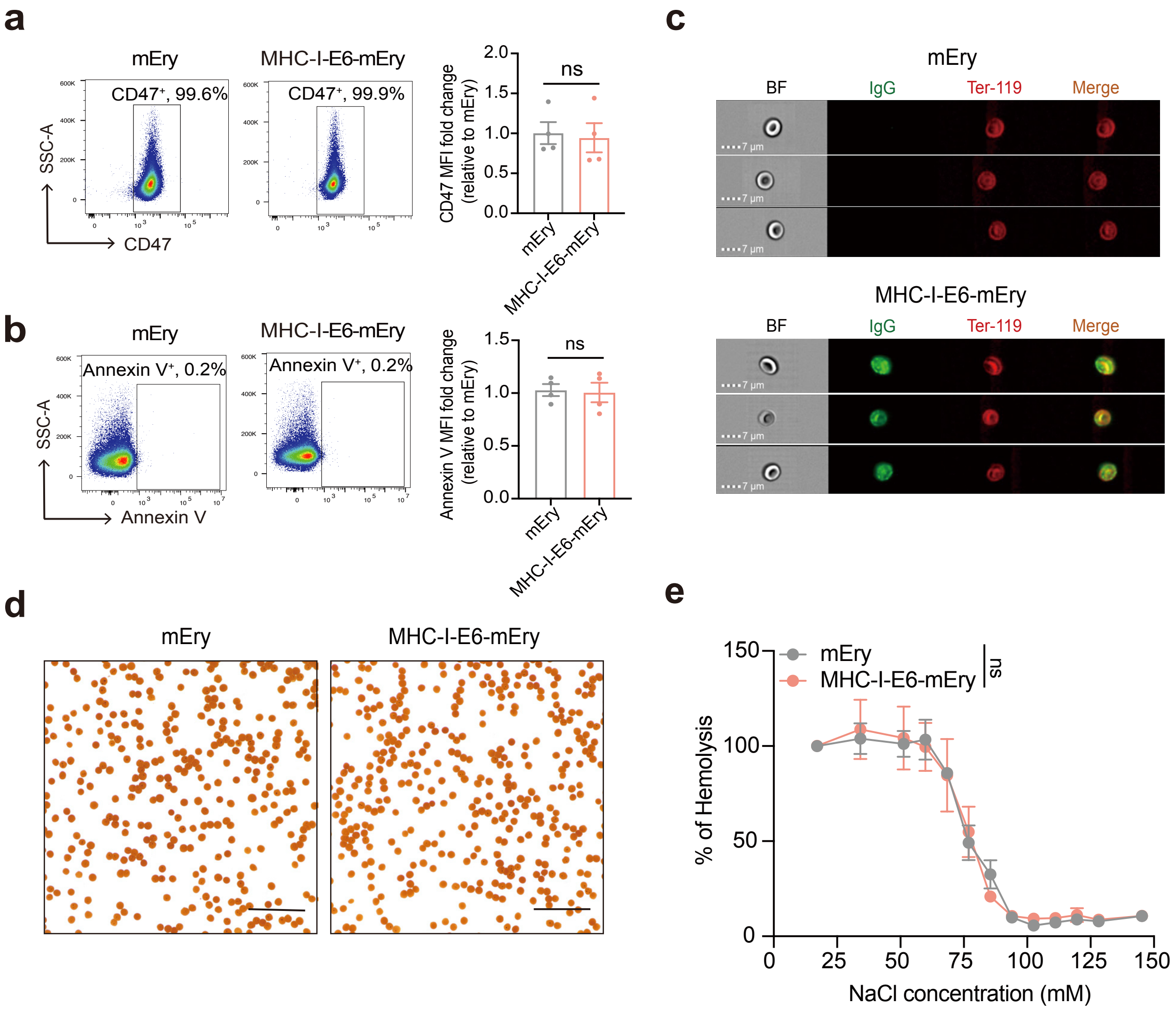

a
mEry
MHC-I-E6-mEry
CD47⁺, 99.6%
CD47⁺, 99.9%
SSC-A
CD47
ns
CD47 MFI fold change (relative to mEry)
mEry
MHC-I-E6-mEry
b
Annexin V⁺, 0.2%
Annexin V⁺, 0.2%
Annexin V
Annexin V MFI fold change (relative to mEry)
c
BF
IgG
Ter-119
Merge
7 µm
d
e
% of Hemolysis
NaCl concentration (mM)

Fig. S2

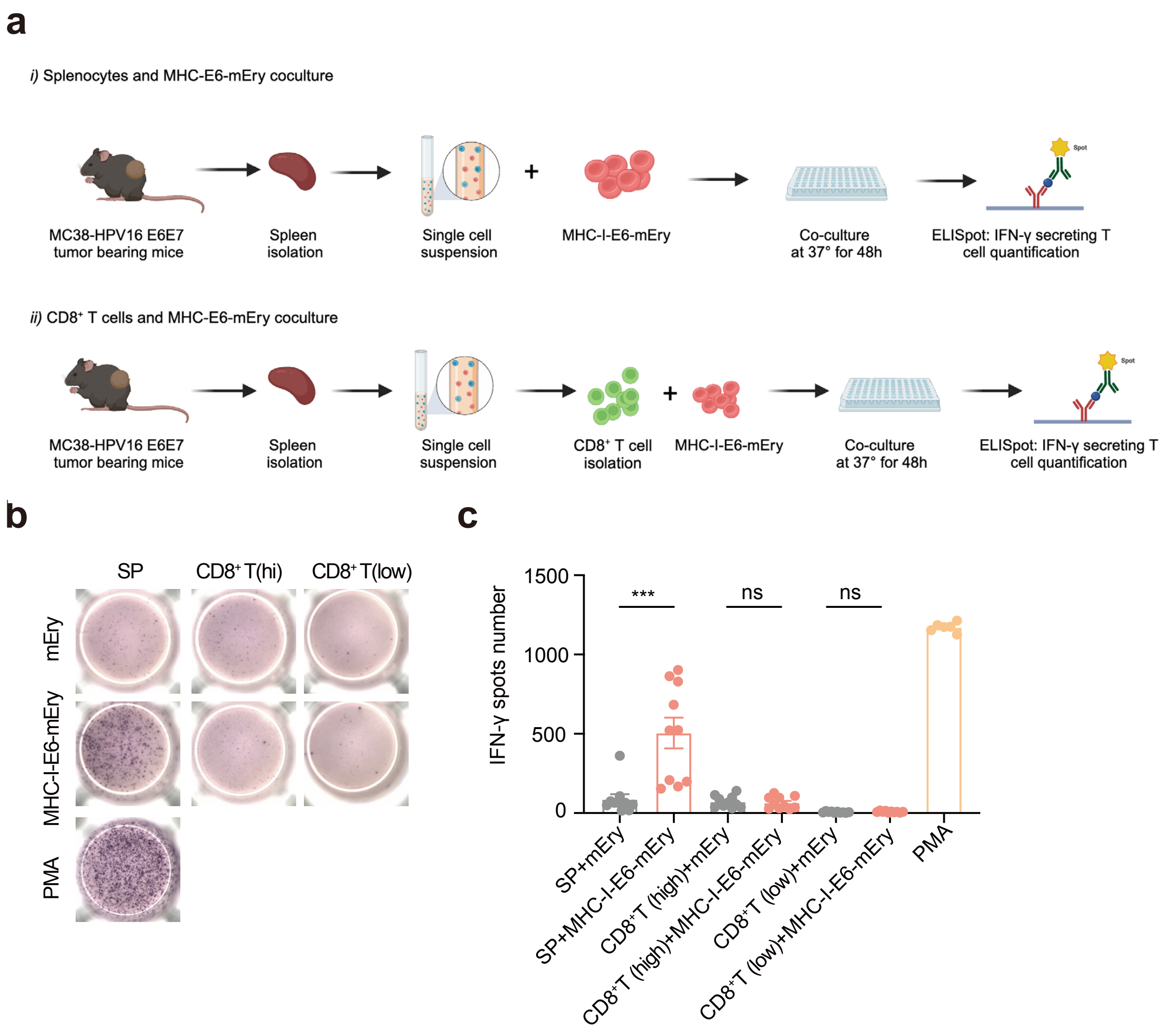
a
i) Splenocytes and MHC-E6-mEry coculture
MC38-HPV16 E6E7 tumor bearing mice
Spleen isolation
Single cell suspension
+
MHC-I-E6-mEry
Co-culture at 37° for 48h
ELISpot: IFN-γ secreting T cell quantification
Spot
ii) CD8+ T cells and MHC-E6-mEry coculture
MC38-HPV16 E6E7 tumor bearing mice
Spleen isolation
Single cell suspension
CD8+ T cell isolation
+
MHC-I-E6-mEry
Co-culture at 37° for 48h
ELISpot: IFN-γ secreting T cell quantification
Spot
b
SP
CD8+ T(hi)
CD8+ T(low)
mEry
MHC-I-E6-mEry
PMA
c
IFN-γ spots number
1500
1000
500
0
***
ns
ns
SP+mEry
SP+MHC-I-E6-mEry
CD8+T (high)+mEry
CD8+T (high)+MHC-I-E6-mEry
CD8+T (low)+mEry
CD8+T (low)+MHC-I-E6-mEry
PMA

Fig. S3

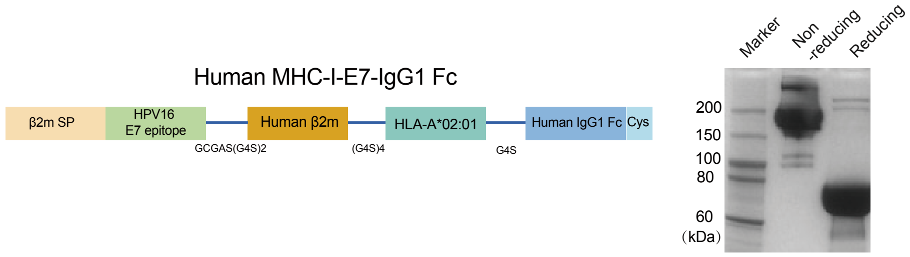
Human MHC-I-E7-IgG1 Fc
β2m SP
HPV16
E7 epitope
Human β2m
HLA-A*02:01
Human IgG1 Fc
Cys
GCGAS(G4S)2
(G4S)4
G4S
Marker
Non
-reducing
Reducing
200
150
100
80
60
(kDa)

Fig. S4

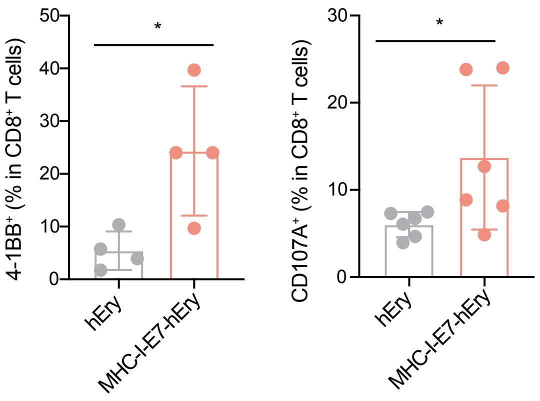

4-1BB⁺ (% in CD8⁺ T cells)
50
40
30
20
10
0
*
hEry
MHC-I-E7-hEry
CD107A⁺ (% in CD8⁺ T cells)
30
20
10
0
*
hEry
MHC-I-E7-hEry

Fig. S5

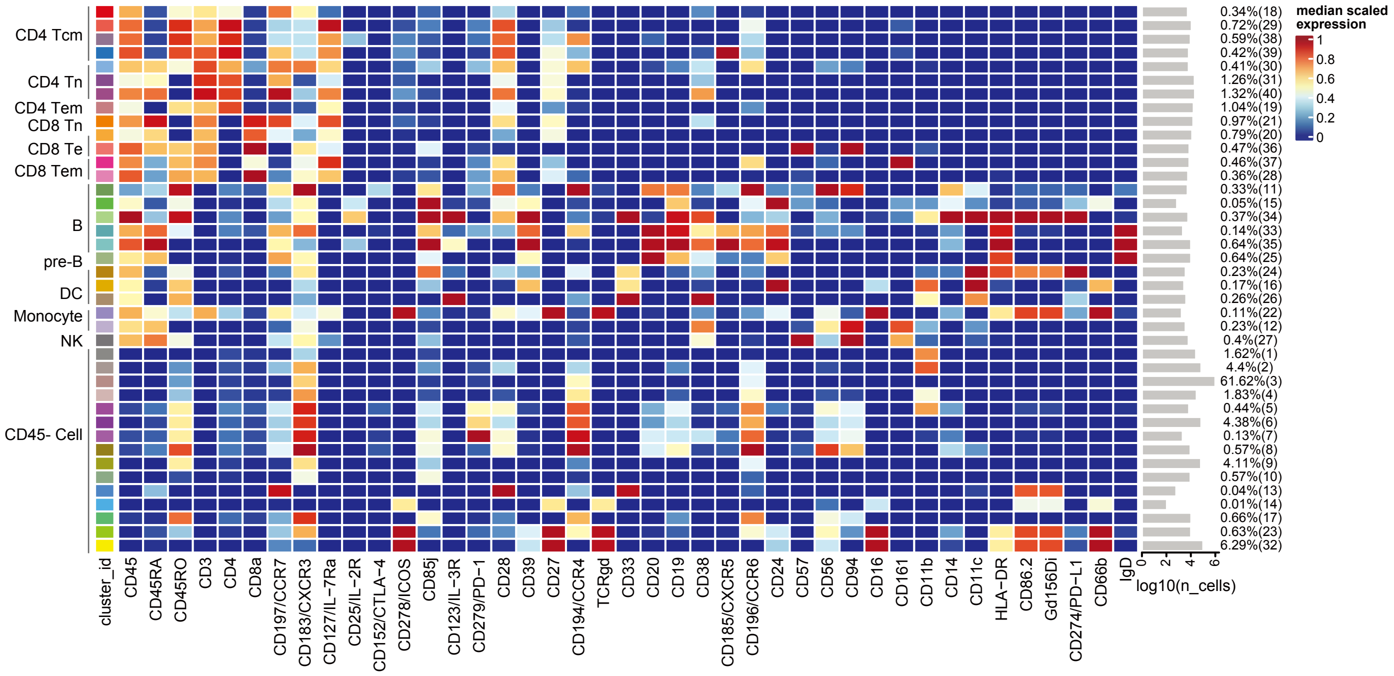

CD4 Tcm
CD4 Tn
CD4 Tem
CD8 Tn
CD8 Te
CD8 Tem
B
pre-B
DC
Monocyte
NK
CD45- Cell
cluster_id
CD45
CD45RA
CD45RO
CD3
CD4
CD8a
CD197/CCR7
CD183/CXCR3
CD127/IL-7Ra
CD25/IL-2R
CD152/CTLA-4
CD278/ICOS
CD85j
CD123/IL-3R
CD279/PD-1
CD28
CD39
CD27
CD194/CCR4
TCRgd
CD33
CD20
CD19
CD38
CD185/CXCR5
CD196/CCR6
CD24
CD57
CD56
CD94
CD16
CD161
CD11b
CD14
CD11c
HLA-DR
CD86.2
Gd156Di
CD274/PD-L1
CD66b
IgD
0 2 4 6
log10(n_cells)
0.34%(18)
0.72%(29)
0.59%(38)
0.42%(39)
0.41%(30)
1.26%(31)
1.32%(40)
1.04%(19)
0.97%(21)
0.79%(20)
0.47%(36)
0.46%(37)
0.36%(28)
0.33%(11)
0.05%(15)
0.37%(34)
0.14%(33)
0.64%(35)
0.64%(25)
0.23%(24)
0.17%(16)
0.26%(26)
0.11%(22)
0.23%(12)
0.4%(27)
1.62%(1)
4.4%(2)
61.62%(3)
1.83%(4)
0.44%(5)
4.38%(6)
0.13%(7)
0.57%(8)
4.11%(9)
0.57%(10)
0.04%(13)
0.01%(14)
0.66%(17)
0.63%(23)
6.29%(32)
median scaled expression
1
0.8
0.6
0.4
0.2
0

Fig. S6

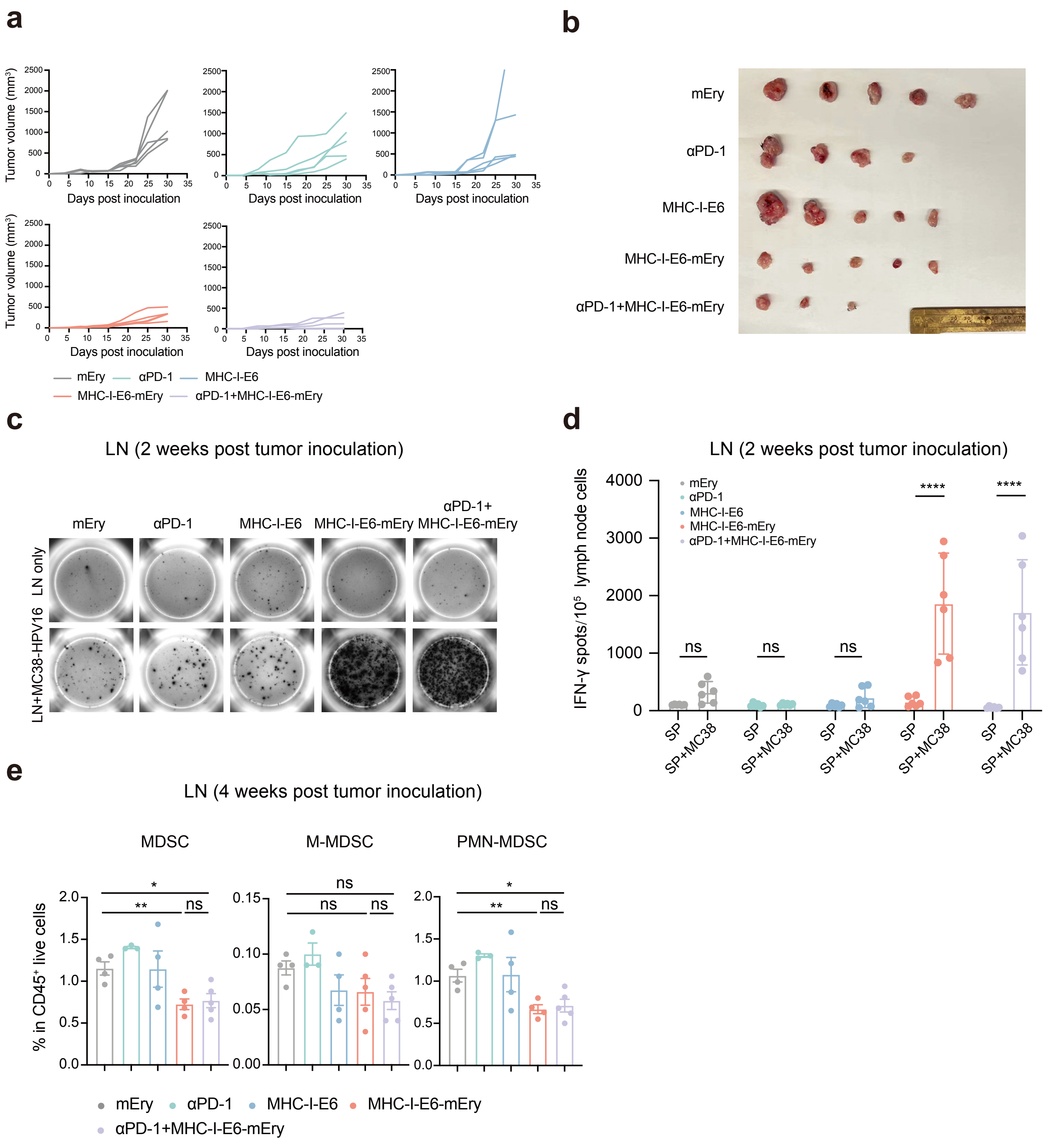
a
Tumor volume (mm³)
Days post inculation
Days post inoculation
mEry
αPD-1
MHC-I-E6
MHC-I-E6-mEry
αPD-1+MHC-I-E6-mEry
b
mEry
αPD-1
MHC-I-E6
MHC-I-E6-mEry
αPD-1+MHC-I-E6-mEry
c
LN (2 weeks post tumor inoculation)
mEry
αPD-1
MHC-I-E6
MHC-I-E6-mEry
αPD-1+
MHC-I-E6-mEry
LN only
LN+MC38-HPV16
d
LN (2 weeks post tumor inoculation)
IFN-γ spots/10⁵ lymph node cells
ns
****
SP
SP+MC38
e
LN (4 weeks post tumor inoculation)
MDSC
M-MDSC
PMN-MDSC
% in CD45⁺ live cells
*
**
ns

Fig. S7

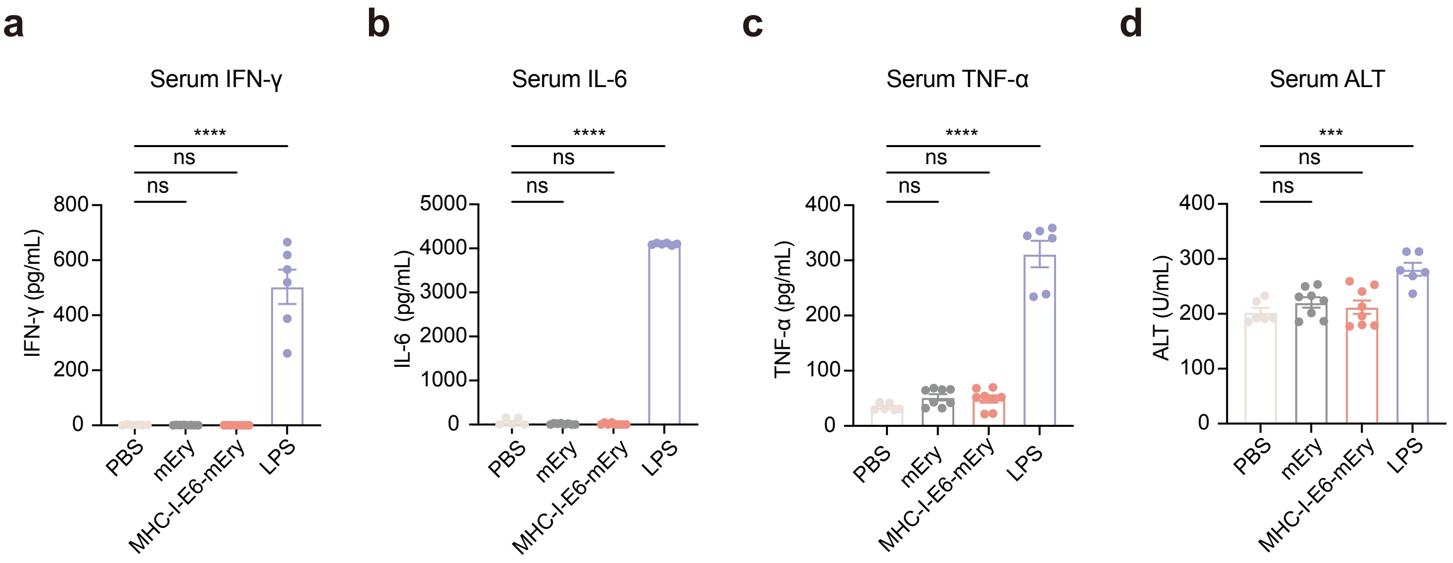

a
Serum IFN-γ
IFN-γ (pg/mL)
****
ns
ns
0
200
400
600
800
PBS
mEry
MHC-I-E6-mEry
LPS
b
Serum IL-6
IL-6 (pg/mL)
****
ns
ns
0
1000
2000
3000
4000
5000
PBS
mEry
MHC-I-E6-mEry
LPS
c
Serum TNF-α
TNF-α (pg/mL)
****
ns
ns
0
100
200
300
400
PBS
mEry
MHC-I-E6-mEry
LPS
d
Serum ALT
ALT (U/mL)
***
ns
ns
0
100
200
300
400
PBS
mEry
MHC-I-E6-mEry
LPS

Fig. S8

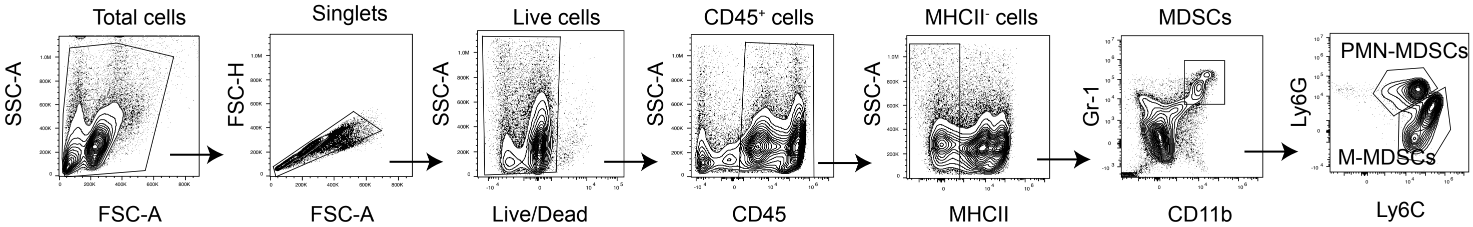
Total cells
SSC-A
FSC-A
Singlets
FSC-H
FSC-A
Live cells
SSC-A
Live/Dead
CD45+ cells
SSC-A
CD45
MHCII- cells
SSC-A
MHCII
MDSCs
Gr-1
CD11b
PMN-MDSCs
M-MDSCs
Ly6G
Ly6C

Fig. S9

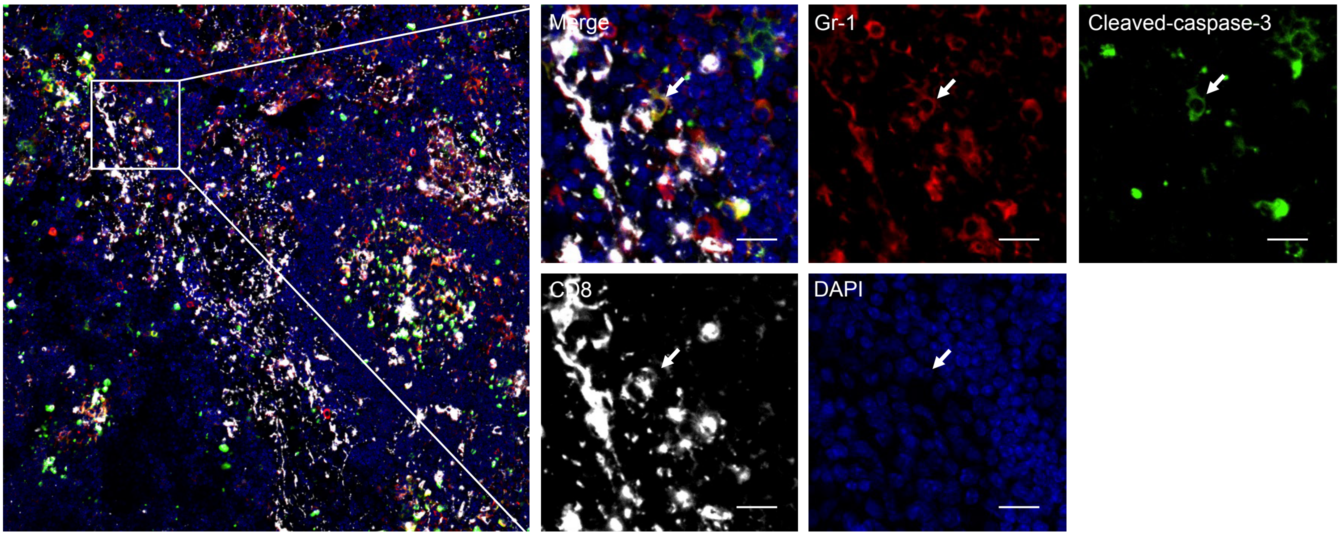

Fig. S10

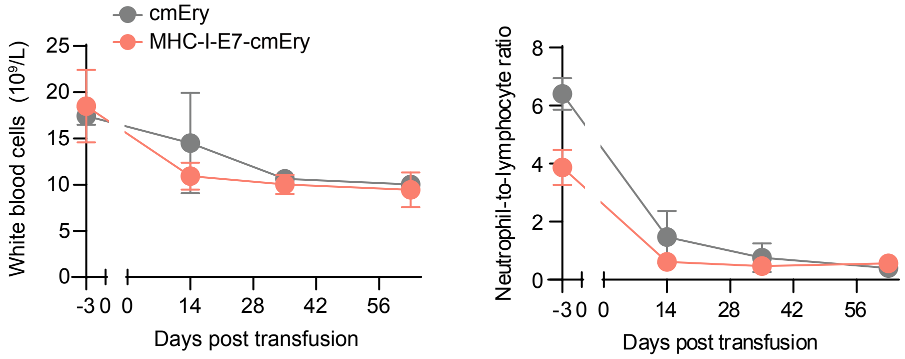

cmEry
MHC-I-E7-cmEry
White blood cells (10⁹/L)
25
20
15
10
5
0
-3 0 0 14 28 42 56
Days post transfusion
Neutrophil-to-lymphocyte ratio
8
6
4
2
0
-3 0 0 14 28 42 56
Days post transfusion

Fig. S11

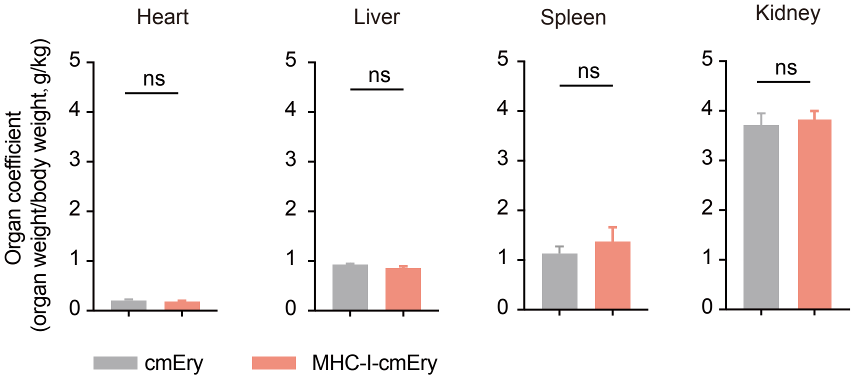
Heart
Liver
Spleen
Kidney
Organ coefficient
(organ weight/body weight, g/kg)
ns
ns
ns
ns
0
1
2
3
4
5
cmEry
MHC-I-cmEry

Fig. S12

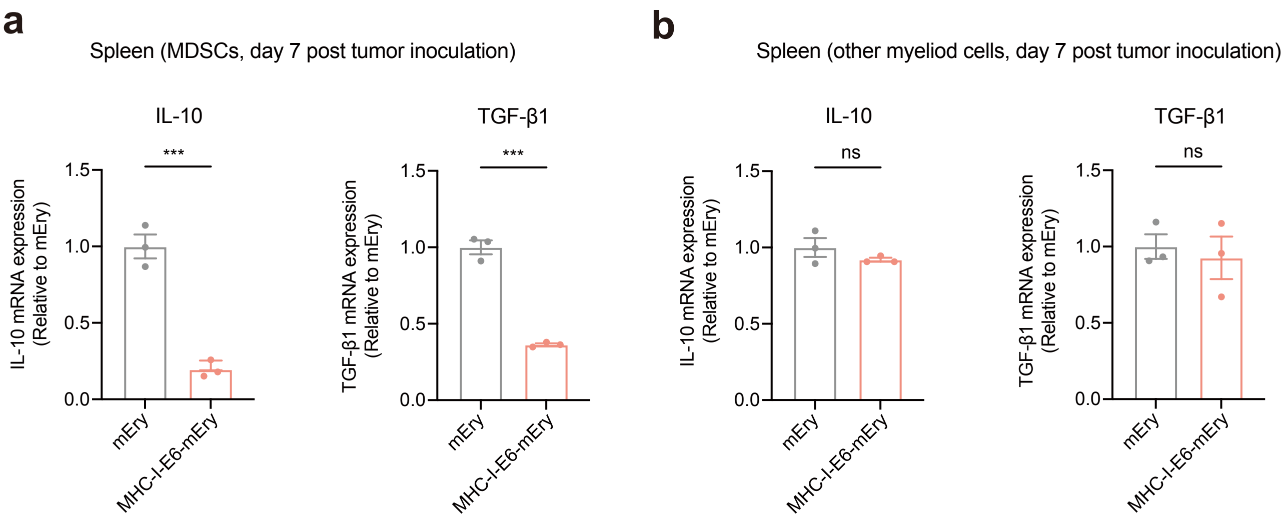

a
Spleen (MDSCs, day 7 post tumor inoculation)
IL-10
***
IL-10 mRNA expression
(Relative to mEry)
1.5
1.0
0.5
0.0
mEry
MHC-I-E6-mEry
TGF-β1
***
TGF-β1 mRNA expression
(Relative to mEry)
b
Spleen (other myeliod cells, day 7 post tumor inoculation)
IL-10
ns
TGF-β1
ns

Supplementary Fig. S1 Conjugation of MHC-I–E6 protein to erythrocytes does not affect the morphology and functionality of erythrocytes. **a** Flow cytometry analysis of CD47 expression on MHC-I–E6–mEry and mEry (*n* = 4 per group). **b** Flow cytometry analysis of PS externalization on MHC-I–E6–mEry and mEry (*n* = 4 per group). **c** Imaging flow cytometry analysis of mEry and αPD-1-mEry. **d** MHC-I–E6–mEry and mEry samples were stained with Benzidine and Giemsa. Erythrocytes are stained brown, while other nucleated cells are stained pink, purple, or light purple. **e** Osmotic fragility curves of MHC-I–E6–mEry and mEry were determined using a hypotonic dilution method (*n* = 4 per group). Data are presented as means ± SEM. Significance was determined using either unpaired *t*-test (**a, b**) or two-way ANOVA with Dunnett's multiple comparison test (**e**). The significance levels are indicated as follows: ns, not significant.

Supplementary Fig. S2 $CD8^+$ T cells cultured alone with MHC-I–E6–mEry do not activate antigen-specific $CD8^+$ T cells. a Schematic representation of in vitro activation of antigen-specific T cells. For the co-culture of splenocytes (SP) with MHC-I–E6–mEry group, splenocytes were collected from MC38-HPV16 tumor-bearing mice and co-incubated with mEry or MHC-I–E6–mEry for 48 h before performing the ELISpot assay. For the co-culture of $CD8^+$ T cells with MHC-I–E6–mEry group, splenic $CD8^+$ T cells were isolated from MC38-HPV16 tumor-bearing mice and then co-incubated with mEry or MHC-I–E6–

mEry for 48 h before performing the ELISpot assay. **b** Representative images of ELISpot wells. **c** Quantitative analysis of IFN-γ spot counts in **(b)**. (*n* = 5 mice per group except for *n* = 3 mice for the PMA group, 2 replicates per mouse). Data are presented as means ± SEM. Significance was determined by unpaired t-test (**c**). ns, not significant; ***P<0.01.

Supplementary Fig. S3 MHC-I–E7–IgG1 Fc fusion protein purification. Left: Diagram illustrating the human MHC-I–E7–IgG1 Fc construct. Right: SDS-PAGE analysis of the purified human MHC-I–E7–IgG1 Fc fusion proteins.

Supplementary Fig. S4 In vitro function of MHC-I–E7–hEry. Expression of 4-1BB and CD107a on $CD8^+$ T cells from CESC patients after the treatment of hEry or MHC-I–E7–hEry were analyzed by flow cytometry. Data are presented as means ± SEM. Significance was determined by unpaired *t*-test. The significance levels are indicated as follows: *P < 0.05.

Supplementary Fig. S5 Characterization of immune cell subpopulations from PBMCs by mass cytometry. Marker heatmap used for immune cell clustering shows median expression scaled per marker for cluster annotation.

Supplementary Fig. S6. MHC-I–E6–mEry exerts inhibitory effects on the growth of MC38 tumors. a Tumor growth curves for MC38-HPV16 tumor

model in individual group (n = 5 mice per group). **b** Representative images of tumors after various treatments (n = 5 mice per group). **c** IFN-γ secreting T cells were tested by ELISpot assay. Lymph node cells isolated 2 weeks after tumor inoculation from different groups were co-cultured with MC38-HPV16 tumor cells (n = 3 mice per group and two replicate well per mice). **d** Quantification of spots number in **c**. **e** Percentage of MDSCs and its subsets in the lymph nodes were assessed by flow cytometry 4 weeks after tumor inoculation (mEry, n = 4; αPD-1, n = 3; MHC-I–E6, n = 4; MHC-I-E6–mEry, n = 4; MHC-I–E6–mEry plus αPD-1, n = 5. Data are presented as means ± SEM. Significance was determined by Student's t test (**d**). The significance levels are indicated as follows: ns, not significant; *P < 0.05; **P < 0.01; ****P < 0.0001.

Supplementary Fig. S7 Safety evaluation of MHC-I–E6–mEry in murine tumor models. a-d MC38-HPV16 tumor-bearing C57BL/6 mice were received treatments of PBS, mEry or MHC-I–E6–mEry twice a week for three times the day after tumor inoculation. Serum samples were collected on day 11 for analysis. For the LPS positive control group, mice were intraperitoneally injected with LPS and serum samples were collected 6 h later. **a-c** Concentrations of IFN-γ (**a**), IL-6 (**b**) and TNF-α (**c**) in serum were quantified using by ELISA assay. **d** Level of serum ALT (PBS, n = 3; LPS, n = 3; mEry, n = 4; MHC-I–E6–mEry, n = 4; 2 replicates per mouse). Data are presented as means ± SEM. Significance was determined using one-way ANOVA with

Dunnett's multiple comparison test (**a-d**). The significance levels are indicated as follows: ns, not significant; ***P < 0.001; ****P < 0.0001.

Supplementary Fig. S8 Gating strategy for identification of MDSCs. Representative flow-cytometry gating strategy for determination of MDSC populations in mouse samples.

Supplementary Fig. S9 CD8$^+$ T cells activated by MHC-I–E6–mEry induce the apoptosis of MDSCs in vivo. Spleens from tumor-bearing mice treated with MHC-I–E6–mEry were isolated for immunofluorescence staining. The following markers were used: Gr-1 (red), Cleaved caspase-3 (green), CD8 (white), and DAPI (blue). The scale bar represents 100 μm.

Supplementary Fig. S10 Safety evaluation of MHC-I–E7–cmEry in cynomolgus macaques. White blood cell number and neutrophil to lymphocyte ratio were assessed throughout the experiment in cynomolgus macaques. Data are presented as the means ± SEM.

Supplementary Fig. S11 Organ coefficient analysis of non-human primates. Organ coefficient (organ/body weight ratios) of heart, spleen, liver, and kidney were evaluated at the endpoint. Data are presented as the means

± SEM. Significance was determined by Student's *t* test. The significance levels are indicated as follows: ns, not significant.

Supplementary Fig. S12 MHC-I–E6–mEry suppresses the expression of immunosuppressive cytokine production in splenic myeloid cells. MC38-HPV16 tumor bearing mice received treatments of mEry or MHC-I–E6–mEry twice weekly for two times the day after tumor inoculation. On day 7 post-inoculation, splenocytes were isolated and subjected to flow cytometry for the separation of MDSC population ($CD45^+CD19^-CD4^-CD8^-CD11b^+Gr\text{-}1^+$) and other myeloid cell populations exclude MDSCs ($CD45^+CD19^-CD4^-CD8^-CD11b^+Gr\text{-}1^-$, $CD45^+CD19^-CD4^-CD8^-CD11b^-Gr\text{-}1^+$, $CD45^+CD19^-CD4^-CD8^-CD11b^-Gr\text{-}1^-$). **a** qPCR analysis of IL-10 and TGF-β1 mRNA expression in MDSCs (n = 3 mice per group). **b** qPCR analysis of IL-10 and TGF-β1 mRNA expression in other myeloid cells (n = 3 mice per group). Data are presented as means ± SEM. Significance was determined using unpaired *t*-test (**a, b**). The significance levels are indicated as follows: ns, not significant; ***P < 0.001.
